# Changing Hearts and Plates: The Effect of Animal-Advocacy Pamphlets on Meat Consumption

**DOI:** 10.3389/fpsyg.2021.668674

**Published:** 2021-05-31

**Authors:** Menbere Haile, Andrew Jalil, Joshua Tasoff, Arturo Vargas Bustamante

**Affiliations:** ^1^Department of Economic Sciences, Claremont Graduate University, Claremont, CA, United States; ^2^Department of Economics, Occidental College, Los Angeles, CA, United States; ^3^Department of Health Policy and Management, Fielding School of Public Health, University of California, Los Angeles, Los Angeles, CA, United States

**Keywords:** vegan, animal advocacy, randomized controlled trial, pamphlets, leaflets

## Abstract

Social movements have driven large shifts in public attitudes and values, from anti-slavery to marriage equality. A central component of these movements is moral persuasion. We conduct a randomized-controlled trial of pro-vegan animal-welfare pamphlets at a college campus. We observe the effect on meat consumption using an individual-level panel data set of approximately 200,000 meals. Our baseline regression results, spanning two academic years, indicate that the pamphlet had no statistically significant long-term aggregate effects. However, as we disaggregate by gender and time, we find small statistically significant effects within the semester of the intervention: a 2.4 percentage-point reduction in poultry and fish for men and a 1.6 percentage-point reduction in beef for women. The effects disappear after 2 months. We merge food purchase data with survey responses to examine mechanisms. Those participants who (i) self-identified as vegetarian, (ii) reported thinking more about the treatment of animals or (iii) expressed a willingness to make big lifestyle changes reduced meat consumption during the semester of the intervention. Though we find significant effects on some subsamples in the short term, we can reject all but small treatment effects in the aggregate.

## 1. Introduction

During the twentieth century, animal farming radically transformed from small family farms to large-scale concentrated animal feeding operations (CAFO), also referred to as factory farms (Norwood and Lusk, 2011). According to USDA data, 99% of US farmed animals are raised in CAFOs (Anthis, 2019). Gains in efficiency have come at the expense of the welfare of the animals. For example, pigs are confined for months in crates measuring only 14 square feet, prohibiting virtually all movement including walking and turning around (Norwood and Lusk, 2011). Egg-laying hens are placed in cramped cages with only 67 square inches allotted per bird, less than one 8.5 × 11 inch sheet of paper (93.5 square inches). To prevent aggression in cramped quarters, a half to a third of their highly sensitive beaks are severed, possibly leading to chronic pain (Duncan, 2001; Fraser et al., 2001; Cheng, 2006; Norwood and Lusk, 2011). These practices are standard industry protocol in the U.S.

While measuring the animals' wellbeing directly is not possible, the evidence indicates that farmed animals suffer under these conditions. Confined pigs show signs of extreme stress, such as bar-biting and other repetitive behaviors. They also become unresponsive, remaining passive when splashed with water, poked, or prodded—a likely sign of severe depression (Broom and Johnson, 1993; Broom et al., 1995; Vieuille-Thomas et al., 1995; Marchant and Broom, 1996). The tight confinement imposed on egg-laying hens prevents exercise which leads to osteoporosis and broken bones. As many as 30% of hens have broken bones before slaughter. During forced molting, hens show signs of severe distress, including aggression and stereotyped pacing (Gregory and Wilkins, 1989; Duncan, 2001). Given the vast amount of meat consumption, the scale of the suffering is likely immense. Based on data from the FAO of the United Nations, approximately 70 billion land animals are slaughtered for food every year, with 74% raised in CAFOs (Sanders, 2018).

This treatment of farmed animals violates the principles of many ethical theories, including utilitarian and deontological frameworks (Singer, 1979; Regan, 1983). Amongst ethicists who write on the issue, “there is widespread (though not perfect) consensus that it is generally morally better for the typical North American to eat less factory farmed meat” (Schwitzgebel et al., 2020). This view is consistent with mainstream American attitudes toward factory farms. In a recent survey, based on a representative sample of the U.S. population, a ban on factory farming, slaughterhouses and animal farming garnered substantial support: 49, 47, and 33%, respectively (Anthis, 2017). Other researchers replicated these results using a different sample (Norwood and Murray, 2018). However, support of the system through meat consumption continues, perhaps for a variety of reasons including but not limited to ignorance or neglect of conditions on farms, a lack of perceived individual agency to effect change, the invisibility of the victims, and the challenge of changing one's habits. In that same study, 58% of the sample agreed that “most farmed animals are treated well,” despite the fact that 99% of farmed animals in the U.S. are raised in CAFOs under the aforementioned conditions.

In this paper, we ask whether moral persuasion through pamphlets can lead to changes in behavior, specifically meat consumption. Moral persuasion, or moral suasion, is the use of normative appeals and rhetoric to affect behavior. It has been a centerpiece of many social movements in history. Harriet Beecher Stowe's *Uncle Tom's Cabin* was one of the bestselling books of its day and is widely believed to have changed attitudes against slavery prior to the American Civil War. Martin Luther King Jr., during the 1960's civil rights movement, advocated for a vision of America in which people would not be judged by their appearance but by their character. More recently, advocates for marriage equality have made their case across numerous platforms, using a strategic legal campaign through litigation and legislative advocacy. Increased social acceptability and positive portrayal of LGBT individuals persuaded millions to change their minds on marriage equality (Kowal, 2015). In each case, moral persuasion led to dramatic changes in legal institutions and social norms.

The animal advocacy movement offers unique advantages for investigation. First, it is ongoing with aims that are far from the status quo. Great scope for change remains. Second, the animal advocacy movement persuades individuals to alter their behavior, specifically to avoid eating meat. Behavior change with individual-level panel data allows for high-powered tests. Third, a common method of persuasion in this social movement is the dissemination of pamphlets to pedestrians. This medium affords us the ability to randomly assign moral persuasion at the individual level.

Studying the effect of moral-advocacy pamphlets specifically is interesting in its own right. Pamphlets have been a historically important medium for advocacy. Martin Luther's *95 Theses* instigated the Protestant Reformation, Thomas Paine's pamphlet *Common Sense* popularized the argument for the American Revolution, and Martin Luther King Jr's *Letter from a Birmingham Jail* advanced the campaign for civil rights (Forman, 2017). Pamphlets are also inexpensive to produce and disseminate, and their physicality may capture more attention than some digital mediums.

We set up a table at a college campus and verbally solicited participation. Undergraduate student subjects were randomly assigned to an animal-advocacy or placebo pamphlet. The animal-advocacy pamphlet specifically requested that people refrain from eating meat to improve the wellbeing of farmed animals. The placebo pamphlet made no mention of diet. We estimated the effect of the treatment pamphlet on meal purchases in the college's main dining halls, with an individual-level data set of over 200,000 food purchases. We supplemented the purchase data with a follow-up survey 1 month after disseminating the pamphlet.

Several studies have attempted to measure the effect of animal-welfare pamphlets on meat consumption but all have used self-reports and have been under powered (Animal Charity Evaluators, 2013; Hennessy, 2016; Flens et al., 2018). See Animal Charity Evaluators (2017) and Peacock and Sethu (2017) for reviews. Our paper adds to the growing literature that uses actual consumption data to measure the effect of an intervention designed to reduce meat consumption. To our knowledge, there are only three previous randomized-controlled trials in this area with real meat consumption data in the field. One examines the effect of defaults (Hansen et al., 2019) and the other two examine the effect of education (Jalil et al., 2020; Schwitzgebel et al., 2020). Three other studies conduct field experiments with exogenous variation to estimate the effect of menu manipulation and product placement (Garnett et al., 2019, 2020; Vandenbroele et al., 2019).

Our pamphlet uses an animal welfare message to persuade individuals to reduce their meat consumption. The extent to which such messages are effective is unclear. Many studies conclude that most people do not want to harm sentient beings, but engage in cognitive dissonance. Rothgerber (2020) develops a psychological framework, termed “meat-related cognitive dissonance,” for how individuals evade guilt when their food choices lead to animal harm (e.g., avoiding information, belittling “do-gooders,” denigrating the animals, formulating pro-meat justifications, rejecting responsibility). Based on survey-level evidence, Bastian et al. (2012) find that when told of the suffering animals experience in meat production, people ascribe lower mental attributes to those animals, likely to justify their continued participation in the system. Schröder and McEachern (2004) find that while people generally agree that cruelty toward animals is intolerable, they develop strategies to cope with the harm animals experience due to their demand for meat. By contrast, in a recent review of the literature, Bianchi et al. (2018) conclude that interventions focusing on animal welfare are associated with intentions to consume less meat. Sonoda et al. (2018) provide evidence that consumers care about animal welfare considerations in their food purchases. Schwitzgebel et al. (2020) find that college students, in response to a class on the ethics of eating meat that talks about animal suffering, reduce their actual meat consumption.

We find in the aggregate, looking at the data over a 2-year time period, no statistically significant effects of the treatment pamphlet. We can reject treatment effects of 1.9 percentage points or larger with 95% confidence. Likewise, when we look at treatment effects in the semester of the intervention and the subsequent semester we find no significant effects. It is only when we disaggregate the effect by time and gender, as we specified in our pre-analysis plan, we find statistically significant effects. Men and women change their diets during the semester of the intervention. Men reduce their consumption of poultry and fish by 2.4 percentage points (5.2%) and increase their consumption of vegetarian and vegan meals by 2.3 percentage points (10.6%), roughly the same magnitude. Women decrease their consumption of beef by 1.5 percentage points (13.3%), but weakly increase their consumption of poultry and fish (ns). Though we expected differences by gender, as we found nearly twice the treatment effect from women compared to men in our previous study (Jalil et al., 2020), we did not expect this pattern, nor do we have a good explanation for it. In the long run, the effects are no longer statistically significant.

Our survey data helps to provide additional insight regarding the mechanisms of the intervention. Those participants who (1) self-identified as vegetarian, (2) reported thinking more about the treatment of animals or (3) expressed a willingness to make big lifestyle changes reduced meat consumption during the semester of the intervention. Together, the evidence suggests that the treatment is more effective for those already predisposed toward meat reduction.

Overall, the effects are small, short-lasting, or non-existent. We test for treatment effects with many other subsamples and only find null results. This is presented in our online Supplementary Information document. Given (1) the unexpected and unexplained gender differences, (2) the overall weak treatment effects, and (3) multiple hypothesis testing with mostly null results, we do not have high confidence that a replication study would produce the same pattern of significant findings. We do think that the significant results are still informative when properly contextualized within the larger literature.

On the flip side, pamphleteering is an inexpensive intervention. Given the low costs, the evidence is also insufficient to claim that pamphleteering is cost ineffective. Even very small effect sizes may justify pamphleteering if the cost of disseminating a pamphlet is miniscule.

## 2. Materials and Methods

### 2.1. Experimental Procedures

We recruited undergraduate students at a U.S. college campus. Experimenters positioned themselves at various locations on campus at times with heavy foot traffic, and asked students to participate in a scientific study that involved receiving a pamphlet, being contacted by email for an online survey, and having a chance to win a gift card. Experimenters gave students a short description of the study and a consent form. No mention of meat consumption or animal welfare occurred before consenting. After reading and signing the consent forms, the students received either the treatment or control pamphlet. The experimenters did not discuss the contents of the pamphlet or give further information. In the consent form, in addition to their name and signature, students provided an email address for future contact. In total, 685 students participated. The pamphleteering was conducted over 2-week segments about 1 month after the start of the spring and fall semesters of 2019.

Approximately 1 month after subjects received their pamphlet, we emailed subjects a link to an online Qualtrics survey that took 3–5 min to complete. We incentivized participation in the survey through a random drawing for a $50 Amazon gift card. The participation rate was 49% with 338 subjects completing the survey. People who eat less meat were more likely to select into the survey (please see Supplementary Information for details).

We individually randomized subjects into treatment and control groups based on their student ID. The treatment group received the animal-advocacy pamphlet *Compassionate Choices*, produced by an activist group, Vegan Outreach. The pamphlet discusses the impact of factory farming and the conditions under which farm animals are treated. The pamphlet also contains information on how to eat less meat, i.e., discussions about the health benefits of eating a plant-based diet, meal ideas that contain no animal products, and personal testimonies from people who have made the choice to adopt a vegetarian lifestyle. While this latter information could also influence behavior, the majority of the pamphlet—its salient message—focuses on animal welfare. The barriers to diet change likely include lack of knowledge about the welfare condition of animals in farms, lack of an emotional connection to the suffering of animals, lack of knowledge about health and plant-based diets, and lack of knowledge about easily available plant-based options. The pamphlet attempts to address all of these issues. However, there are likely other barriers to diet change that the pamphlet simply cannot address, such as a long-ingrained habit of meat consumption.

The control group received the pamphlet *The Cruelty Behind the Cuteness*, a pamphlet produced by the Humane Society of the United States. It discusses problems with “puppy mills.” It does not mention diet.

### 2.2. Data

We collected three types of data: food-purchase data, post-intervention Qualtrics survey data, and administrative data on gender. Students swiped their ID card, via their meal plan, to purchase food at the dining facilities. Cashiers chose one of four buttons that register the main entree: beef, poultry, fish, and veg. Vegetarian and vegan (“veg”) meals were always available at every food station, offering students a choice between a plant-based and meat-based dish. Meals were a la carte, allowing us to observe students' food choices. The prices for the meat and veg options were usually the same. We exclude snacks and purchases where cashiers did not differentiate between meat and non-meat options (i.e., Friday evenings, weekday mornings and weekends). We collected data for four consecutive semesters totaling roughly 200,000 meals.

The survey data came from our online Qualtrics survey conducted a month after the intervention for both treatment and control groups. The survey questions asked about participant's demographic information, self-identified current diet, memory recall on the pamphlets, views toward treatment of farm animals, impact of personal choice, attempted diet change, reasons for changing, willingness to make big lifestyle changes, etc. The full details are in the Supplementary Information. We collected administrative data on gender from the card office for study participants. We have registered a research protocol containing the pre-analysis plan for this experiment at the AEA RCT registry with ID AEARCTR-0003871. Our pre-analysis plan is publicly available at www.socialscienceregistry.org/trials/3871.

## 3. Results

### 3.1. Main Treatment Effects

We set up a booth at a U.S. college campus and recruited passing students to participate in an experiment. We randomly assigned subjects to either a placebo or treatment pamphlet. The placebo pamphlet discussed pet adoption and problems with puppy mills. The treatment pamphlet described the conditions of animals at factory farms and made an explicit call to action to adopt a vegan diet. The data set used in our analysis contains approximately 200,000 meal purchases from 685 students. For all subjects, we observe a baseline period prior to the intervention and a post-intervention period, allowing us to estimate within-person changes in diet. Recruitment was ongoing, leading to exogenous variation in the timing of the intervention and helping to control for any seasonal or calendar effects.

Our empirical strategy is to regress food choice on a treated indicator. We categorize all the food items into “beef,” “poultry/fish,” and “vegetarian” (see Methods). The omitted category is “salad bar,” in which students choose from primarily vegetable options, but meat options are also present. Cashiers do not distinguish between salads with and without meat. We omit this category because its contents are ambiguous, however we retain “salad bar” observations in our analysis. We define a fourth category “meat” as containing “beef” or “poultry/fish.” To estimate the average treatment effects of the intervention, we use a difference-in-difference framework. Specifically, we estimate the following logit regression:

(1) $$\begin{array}{lll} \log\left(\frac{F_{m,i,d,h}}{1-F_{m,i,d,h}}\right) & = & \alpha +\beta_{0}T_{m}+\beta_{1}A_{m}+\beta_{2}T_{m}A_{m}\\ +\rho_{i}+\gamma_{d}+\delta_{h}+\varepsilon_{m,i,d,h} \end{array}$$

where *F* is one of four food indicator variables that equals one if the meal purchase, m, belongs to that category (beef, poultry/fish, meat, or veg) for individual i, on day d, at hour h. *T*_*m*_ is an indicator variable for meals purchased by an individual in the treatment group, and *A*_*m*_ is an indicator variable for meals purchased after receiving the pamphlet. The key variable of interest is the interaction term, *T*_m_*A_*m*_, which measures the change in the food outcome variable after receiving the pamphlet for the treatment group, relative to the control group. The interaction term estimates the effects of the pamphlet on participants' food choices. We control for individual (ρ_*i*_), date (γ_*d*_), and hour (δ_*h*_) fixed effects, and cluster standard errors at the individual level. We display all results as average marginal effects.

Table 1 displays the aggregate treatment effect of the animal-advocacy pamphlet. Columns (1)–(4) display the treatment effect over the full sample period 20 August 2018 to 2 June 2020. None of the coefficients are statistically different from zero. In Columns (5)–(8), we show the treatment effect during the semester of the intervention and in Columns (9)–(12) we show the treatment effect in the semesters after the intervention (they include meals purchased before the intervention, and meals purchased after the semester of the intervention). There is no statistically significant effect of the treatment on food choice for any of our outcomes and in any of our time windows.

**Table 1:** Main effect of pamphlet on food consumption.

	**All observations**				**Semester of intervention**				**Semester after intervention**			
	**(1)**	**(2)**	**(3)**	**(4)**	**(5)**	**(6)**	**(7)**	**(8)**	**(9)**	**(10)**	**(11)**	**(12)**
	**Beef**	**Poultry/fish**	**Veg**	**Meat**	**Beef**	**Poultry/fish**	**Veg**	**Meat**	**Beef**	**Poultry/fish**	**Veg**	**Meat**
Treated	–0.002	–0.001	0.003	–0.003	–0.006	–0.004	0.007	–0.010	–0.001	0.002	0.000	0.001
	(0.005)	(0.008)	(0.007)	(0.008)	(0.005)	(0.008)	(0.008)	(0.008)	(0.006)	(0.010)	(0.010)	(0.011)
Mean of DV	0.161	0.394	0.279	0.556	0.158	0.397	0.279	0.555	0.164	0.393	0.276	0.557
PseudoR2	0.116	0.105	0.134	0.151	0.12	0.11	0.137	0.155	0.115	0.101	0.133	0.15
Clusters	681	685	686	685	676	685	686	685	677	684	685	685
*N*	199,716	199,756	199,963	199,835	126,780	127,209	127,394	127,292	156,727	156,962	157,087	157,008

*The coefficents measure logit average marginal treatment effects with standard errors clustered at the individual level for all observations, the semester of intervention and after. Mean DV: mean of the dependent variable. All columns control for individual, date, and hour fixed effects*.

Past research has shown that men and women respond to interventions aimed at diet-change in different ways (Jalil et al., 2020). As we specified in our pre-analysis plan, we estimate the treatment effects disaggregated on men and women in Table 2. We interact the treated indicator with a gender indicator and display the treatment effect by gender. In Columns (1)–(4), during the semester of the intervention, men significantly decrease their consumption of poultry or fish by 2.4 percentage points (5.2%) and increase their consumption of vegetarian/vegan meals by roughly the same magnitude, 2.3 percentage points (10.6%), suggesting substitution from meat to vegetarian/vegan meals. Overall, meat consumption for men falls by the same magnitude as the decline in poultry/fish, 2.4 percentage points (3.6%). Women, in contrast, significantly reduce beef consumption by 1.5 percentage points (12.5%). Poultry and fish consumption increases, though insignificantly, which explains why overall meat consumption does not fall for women. This finding suggests substitution from red meat (beef) to poultry/fish for women. It also explains the lack of detectable effects in Table 1, which does not disaggregate by gender. In Cols (5)–(8) of Table 2, in the semesters following the intervention, none of the effects remain statistically significant.

**Table 2:** Effect of pamphlets by gender.

	**Semester of intervention**				**Semester after intervention**			
	**(1)**	**(2)**	**(3)**	**(4)**	**(5)**	**(6)**	**(7)**	**(8)**
	**Beef**	**Poultry/fish**	**Veg**	**Meat**	**Beef**	**Poultry/fish**	**Veg**	**Meat**
Treated	0.003	–0.024^**^	0.023^*^	–0.024^*^	–0.003	–0.015	0.020	–0.020
	(0.008)	(0.011)	(0.012)	(0.013)	(0.009)	(0.015)	(0.016)	(0.018)
Treated × female	–0.019^*^	0.037^**^	–0.025^*^	0.023	0.004	0.029	–0.029	0.034
	(0.010)	(0.016)	(0.014)	(0.016)	(0.013)	(0.020)	(0.019)	(0.021)
*P*-value female	0.040	0.235	0.864	0.959	0.932	0.262	0.432	0.282
Mean of untreated DV female = 0	0.208	0.462	0.217	0.67	0.218	0.456	0.214	0.674
Mean of untreated DV female = 1	0.12	0.349	0.325	0.47	0.125	0.347	0.321	0.472
PseudoR2	0.12	0.111	0.137	0.155	0.115	0.102	0.133	0.15
Clusters	676	685	686	685	677	684	685	685
*N*	126,780	127,209	127,394	127,292	156,727	156,962	157,087	157,008

*The coefficents measure logit average marginal treatment effects with standard errors clustered at the individual level for the semester of intervention and after the semester of intervention. Mean DV: mean of the dependent variable. All columns control for individual, date, and hour fixed effects*.

* *p < 0.1*;

** *p < 0.05*;

***p < 0.01.

We find statistically significant effects by gender within the semester of the intervention, but not afterwards. Table 3 examines the time path of this effect more closely by breaking apart the treatment effect into three time-windows: the month after the intervention, the second month after the intervention, and later. All columns use the same pre-intervention period, i.e., all meals purchased before the pamphleteering, but restrict the post-intervention period to different windows: the first month after the intervention in Cols (1)–(4), the second month after the intervention in Cols (5)–(8), and afterwards in Cols (9)–(12). The results show no significant effects in the first month after the intervention. Instead, the reductions in beef and poultry/fish for men and women, respectively, are statistically significant in the second month after the intervention. While those coefficients are negative in the first month, they are larger in magnitude and only become significant in the second month.

**Table 3:** Effect of pamphlets by gender–first month, second month, after.

	**First month**				**Second month**				**After 2 months**			
	**(1)**	**(2)**	**(3)**	**(4)**	**(5)**	**(6)**	**(7)**	**(8)**	**(9)**	**(10)**	**(11)**	**(12)**
	**beef**	**Poultry/fish**	**Veg**	**Meat**	**Beef**	**Poultry/fish**	**Veg**	**Meat**	**Beef**	**Poultry/fish**	**Veg**	**Meat**
Treated	–0.004	–0.018	0.024	–0.025	0.014	–0.026^**^	0.018	–0.014	–0.003	–0.015	0.018	–0.021
	(0.009)	(0.014)	(0.015)	(0.016)	(0.009)	(0.013)	(0.014)	(0.014)	(0.008)	(0.014)	(0.015)	(0.017)
Treated × female	–0.005	0.023	–0.022	0.024	–0.032^***^	0.045^**^	–0.022	0.014	0.001	0.032^*^	–0.028	0.035^*^
	(0.013)	(0.018)	(0.017)	(0.019)	(0.012)	(0.019)	(0.017)	(0.019)	(0.012)	(0.019)	(0.018)	(0.020)
*P*-value female	0.322	0.662	0.881	0.895	0.045	0.180	0.739	0.984	0.827	0.168	0.406	0.249
Mean of untreated DV female = 0	0.212	0.459	0.214	0.671	0.21	0.461	0.216	0.671	0.217	0.457	0.215	0.675
Mean of untreated DV female = 1	0.124	0.346	0.326	0.47	0.119	0.352	0.321	0.471	0.125	0.347	0.321	0.472
PseudoR2	0.121	0.109	0.138	0.154	0.12	0.108	0.135	0.154	0.115	0.102	0.134	0.15
Clusters	675	685	685	685	671	685	685	684	677	685	685	685
*N*	106,331	106,847	106,970	106,892	104,071	104,629	104,757	104,677	159,585	159,856	159,947	159,869

*The coefficients measure logit average marginal treatment effects with standard errors clustered at the individual level. The estimation is split in to three periods after the intervention; first month, second month, and after 2 months. Mean DV: mean of the dependent variable. All columns control for individual, date, and hour fixed effects*.

* *p < 0.1*;

** *p < 0.05*;

*** *p < 0.01*.

### 3.2. Heterogeneous Treatment Effects Using Survey Data

Our survey questions help to uncover the mechanisms behind the intervention. We first explore whether the intervention had heterogenous effects as a function of diet. Self-identified vegetarians may have already wrestled with the ethical issues of meat consumption in the past and been more receptive to accept the message. We find that self-reported vegetarians actually purchase a non-negligible fraction of their meals as meat: approximately 17.5% for untreated observations (the control group and pre-intervention treatment group). This finding suggests that while self-identified vegetarians may strive to reduce their meat consumption, they may not be successful at eliminating it from their diet.

We test whether the pamphlet had a significant effect on the fraction of individuals who identify as vegetarian. One month after receiving the pamphlet, the survey asked participants to self-report their diet. We find no significant difference between the control (17.8%) and treatment (12.1%) conditions in the fraction of survey takers who report being vegetarian (see Supplementary Information for details). Because the randomization of individuals into control and treatment groups should have led to roughly equal percentages of vegetarians in both groups pre-pamphleteering, this finding of similar percentages post-pamphleteering suggests that the pamphlet did not cause a significant increase in self-identified vegetarianism in the treatment group. However, in Table 4, we interact an indicator variable for self-reported vegetarians with the treated indicator to estimate heterogenous treatment effects. Table 4 shows that self-reported vegetarians strongly reduce their poultry/fish consumption in the first month by 13.1 percentage points—effectively reducing their consumption of poultry/fish nearly to zero. Their overall meat consumption also decreases by 9.9 percentage points (56.5%), though it is not statistically significant. Over longer time windows the treatment effect on poultry/fish becomes non-significant.

**Table 4:** Heterogeneous effects by veg identification.

	**First month**				**Second month**				**After 2 months**			
	**(1)**	**(2)**	**(3)**	**(4)**	**(5)**	**(6)**	**(7)**	**(8)**	**(9)**	**(10)**	**(11)**	**(12)**
	**Beef**	**Poultry/fish**	**Veg**	**Meat**	**Beef**	**Poultry/fish**	**Veg**	**Meat**	**Beef**	**Poultry/fish**	**Veg**	**Meat**
Treated	–0.007	0.004	0.013	–0.001	–0.007	0.009	–0.007	0.003	–0.008	–0.004	0.000	–0.012
	(0.010)	(0.013)	(0.014)	(0.014)	(0.009)	(0.014)	(0.014)	(0.015)	(0.008)	(0.014)	(0.015)	(0.016)
Treated × vegn	0.006	–0.135^**^	0.049	–0.098	0.001	–0.002	0.022	–0.001	0.025	0.088	–0.004	0.082
	(0.040)	(0.068)	(0.040)	(0.061)	(0.048)	(0.079)	(0.038)	(0.061)	(0.035)	(0.087)	(0.041)	(0.061)
*P*-value vegn	0.984	0.051	0.095	0.095	0.893	0.928	0.673	0.977	0.603	0.331	0.913	0.238
Mean of untreated DV vegn = 0	0.168	0.418	0.252	0.586	0.165	0.423	0.249	0.587	0.172	0.418	0.254	0.59
Mean of untreated DV vegn = 1	0.0515	0.124	0.585	0.175	0.0505	0.125	0.584	0.175	0.0491	0.119	0.591	0.168
PseudoR2	0.13	0.124	0.154	0.167	0.131	0.122	0.154	0.167	0.126	0.117	0.154	0.167
Clusters	336	342	342	343	335	342	342	342	339	342	342	343
*N*	51,830	52,174	52,203	52,237	51,233	51,540	51,581	51,595	77,377	77,633	77,579	77,661

*The coefficents measure logit average marginal treatment effects with standard errors clustered at the individual level. Vegn refers to self - reported vegetarians. The estimation is split in to three periods after the intervention; first month, second month, and after 2 months. Mean DV: mean of the dependent variable. All columns control for individual, date, and hour fixed effects*.

* *p < 0.1*;

** *p < 0.05*;

*** *p < 0.01*.

Our survey provides additional variables about the mechanism of action. Interacting the treatment with these variables can help reveal the role of various mechanisms. The full analysis is in the Supplementary Information. Here we report the significant findings. We find one mechanism variable that predicts lower treatment effects. A survey question asked, “Reading the leaflet(s) taught me about (choose all the reasons that apply).” *Taughtme* takes the value one if the subject clicked the radio button, “The treatment of animals in farms.” In Table 5, we interact this indicator with the treated indicator. Those who stated that the pamphlet taught them about the mistreatment of farm animals exhibited no change in meat consumption while those who stated that they were not taught by the pamphlet significantly decreased their meat consumption by 3.6 percentage points (7.2%) in the first month. The effect is no longer significant after the first month.

**Table 5:** Heterogenous effects by survey measures: “taught me,” “thought more,” “willingness to make a change.”

	**First month**	**Second month**	**After 2 months**
	**(1)**	**(2)**	**(3)**
	**Meat**	**Meat**	**Meat**
Treated	–0.036^**^	0.010	–0.001
	(0.017)	(0.021)	(0.024)
Treated × taughtme	0.051	–0.026	–0.026
	(0.033)	(0.031)	(0.041)
*P*-value taughtme	0.602	0.471	0.441
Mean of untreated DV taughtme = 0	0.499	0.502	0.497
Mean of untreated DV taughtme = 1	0.524	0.527	0.53
PseudoR2	0.196	0.195	0.188
Clusters	209	208	209
*N*	31,922	31,841	47,386
Treated	–0.015	0.042^*^	0.012
	(0.018)	(0.022)	(0.026)
Treated × thoughtmore	–0.013	–0.084^***^	–0.061
	(0.030)	(0.030)	(0.042)
*P*-value thoughtmore	0.250	0.034	0.135
Mean of untreated DV thoughtmore = 0	0.53	0.531	0.522
Mean of untreated DV thoughtmore = 1	0.486	0.489	0.498
PseudoR2	0.192	0.191	0.185
Clusters	214	213	214
*N*	32,860	32,743	48,456
Treated	–0.053	0.120	0.075^*^
	(0.120)	(0.089)	(0.039)
Treated × smallchange	0.066	–0.124	–0.094^**^
	(0.116)	(0.099)	(0.047)
Treated × moderatechange	0.035	–0.113	–0.058
	(0.119)	(0.098)	(0.045)
Treated × bigchange	–0.075	–0.232^**^	–0.155
	(0.167)	(0.099)	(0.113)
*P*-value smallchange	0.437	0.865	0.452
*P*-value moderatechange	0.359	0.739	0.434
*P*-value bigchange	0.273	0.027	0.462
Mean of untreated DV nochange	0.584	0.583	0.587
Mean of untreated DV smallchange	0.638	0.638	0.633
Mean of untreated DV moderatechange	0.488	0.487	0.494
Mean of untreated DV bigchange	0.148	0.158	0.163
PseudoR2	0.169	0.168	0.166
Clusters	334	333	334
*N*	50,694	50,121	75,505

*The coefficents measure logit average marginal treatment effects with standard errors clustered at the individual level. The variable meat = beef + poultry + fish purchases. Mechanisms: Leaflet taught me about treatment of animals in farms, I thought more about treatment of animals in farms and willigness for change. The estimation is split in to three periods after the intervention; first month, second month, and after 2 months. Mean DV: mean of the dependent variable. All columns control for individual, date, and hour fixed effects*.

* *p < 0.1*;

** *p < 0.05*;

*** *p < 0.01*.

We find two variables that are significantly associated with the treatment effect: (1) thought more and (2) willing to make big lifestyle changes. A survey question stated, “After reading the leaflet I thought more about (choose all the reasons that apply),” and if the subject checked, “The treatment of animals in farms,” then we code the indicator *thoughtmore* as one, otherwise it is zero. This variable is designed to identify those individuals who read the pamphlet and reported thinking more about the treatment of farm animals. Another survey question asked, “How willing are you to make lifestyle changes to help reduce mistreatment of farm animals?” The options were, “Not willing to make any lifestyle changes,” “Willing to make small lifestyle changes,” “Willing to make moderate lifestyle changes,” and “Willing to make big lifestyle changes.” This question identifies the degree to which individuals report a willingness to change their behavior.

Table 5 shows that subjects who indicated thinking more about the treatment of farm animals significantly decreased their consumption of meat. In the second month, the decrease in meat consumption is 4.3 percentage points (8.8%) for this group and significant at the *p* = 0.034. Turning to the “willingness for change question,” Table 5 shows that those who state they are willing to make a big change significantly decreased their meat consumption in the second month by 11.2 percentage points (70.9%) (*p* = 0.027). Again the effect occurs not immediately, but rather in the second month.

These results should be interpreted with caution. In the Supplementary Tables A.5–A.17, we test 17 mechanism variables, including the variables in Table 5, for heterogeneous treatment effects each over 3 time windows. Limiting ourselves to only the meat outcome yields 51 tests. By luck, some of these tests are expected to be significant. To correct for multiple hypothesis tests, we compute sharpened False Discovery Rate (FDR) *q*-values. These can be interpreted as *p*-values corrected for multiple-hypothesis testing. We use the method of Benjamini et al. (2006) as presented in Anderson (2008). None of the *q*-values are below 0.1, suggesting that the mechanism results are unlikely to replicate.

## 4. Discussion

The results show that the animal-advocacy pamphlets had no detectable aggregate effects in the short or long term. We are able to reject treatment effects of reducing meat in the first semester by 2.6 percentage points or larger (*CI* = [−0.026, 0.006]), in the second semester by 2.1 percentage points or larger (*CI* = [−0.021, 0.023]), and over both semesters by 1.9 percentage points or larger (*CI* = [−0.019, 0.013]), with 95% confidence. Moreover, the method of distributing the pamphlet (i.e., asking participants to sign a consent form and then weeks later, complete a survey) may have led to greater engagement with the pamphlet than what would have occurred outside of the setting of a study. As such, the effects we observe may be larger than the true effects in a real-world context. We can reject all but small treatment effects in the aggregate.

Disaggregating by time and gender, we find that men significantly reduce their poultry and fish consumption and women significantly reduce their beef consumption but only during the semester of the intervention. Whereas, men reduced overall meat consumption by switching from poultry and fish toward vegetarian and vegan meals, women appear to have switched from beef toward poultry and fish, suggesting a substitution effect in terms of the types of meat purchased^1^. Past research shows that interventions designed to reduce meat consumption may have different effects on men and women. Jalil et al. (2020) find that a classroom intervention on the climate-change impact of food choices reduced meat consumption nearly twice as much for women as for men. We again find that men and women react to the intervention in different ways. Given that men and women have dramatically different baseline diets, it is natural that treatment effects differ between the two groups. In our study, before the intervention, men consume approximately 75% more beef, 30% more poultry/fish, 35% less vegetarian meals, and 40% more total meat than women. Ex ante, we expected to again see larger treatment effects for women than for men. The pattern that we instead observe is unexpected and not easily explained by past results or differences in initial consumption patterns. Furthermore, these treatment effects are temporary, manifesting not immediately, but rather in the second month after the intervention. The unexplained pattern and delayed onset, combined with the marginal significance (*p*-values for the estimated treatment effects never go below 0.01), and short-lasting effects cast doubt that these results would replicate.

If the treatment effects are real, why were they short-lived? Reducers may have exhausted their motivation, found vegetarian/vegan options to be inconvenient or less desirable, or forgot their intention as old habits took over. Habit change is hard and many interventions designed to change habits fail (see for example Carden and Wood, 2018). Alternatively, the winter and summer vacations may have played a disruptive role. In between semesters, students usually go back home. Sustaining a new diet while living away from campus could be challenging. The vacations may be culpable, coinciding with the attenuation of the treatment effects by gender.

Self-identified vegetarians experience very large changes in diet, though the standard errors are also large. Self-identified vegetarians consume about 17.5% of their meals with meat, with more than two-thirds of those meat-based meals containing poultry/fish. Why are “vegetarians” eating meat in the first place? The vegetarian diet may be aspirational, new or temporary, vegetarians may slip, or vegetarians may purchase some meals for their friends. In any case, the treatment reduces poultry/fish consumption by 13.1 percentage points, effectively eliminating it in the first month. Overall meat consumption reduces by 9.9 percentage points (57%), though it is not significant at conventional levels (*p* = 0.095). Whereas, those who eat a large fraction of their meals with meat have much greater capacity to reduce their consumption, self-identified vegetarians, who are already eating much smaller amounts of meat, reduce the most in response to the pamphlets. This finding suggests that, to the extent that the treatment had any effect, it may have primarily affected people who were already aligned with the message. We present more evidence in support of this interpretation below.

We found two mechanism variables that predicted larger treatment effects, and one mechanism variable that paradoxically predicted weaker treatment effects. We caution that these significant effects do not survive sharpened FDR correction. It is quite likely that these correlations reflect sampling error and will not replicate. However, we offer some interpretation on the chance that they reflect real changes. Those who “thought more” about the issue after reading the pamphlet and those who reported a willingness to make “big change” exhibited significant reductions in meat consumption in the second month. These findings suggest that those who engaged more with the pamphlet, i.e., thought more about the ethical issues, were more likely to change their diet. Expression of willingness to make a big change was an effective leading indicator of that change. These two variables may have identified individuals with greater intrinsic motivation to change. Interestingly, those who said that the pamphlet “taught” them about the treatment of animals in farms exhibited no treatment effect, but those who did not click this response did exhibit a treatment effect. We interpret this finding as evidence that the pamphlet affected those who were already aware of the issue, but not those who were previously ignorant—and for whom the pamphlet taught them new information. Jalil et al. (2020) found the same result in their study. The pamphlet appears to have been more effective with people who were already aware of the issue. These findings suggest that those who know the least about an issue may be the least likely to respond to this type of policy intervention.

The “stages of change” model from the field of psychology may explain this finding (Prochaska and DiClemente, 1982, 1983). This model posits an order of stages that a person moves through on the path to behavior change: precontemplation, contemplation, action, and maintenance. The pamphlets may shift some from the precontemplation to the contemplation stage, and others from the contemplation to action stages. Only the latter shift results in behavior change. This theory may help explain why the treatment was effective for some groups—those already at the contemplation stage, i.e., self-identified vegetarians and individuals for whom the information was not new.

Though we are able to reject all but small treatment effects, this does not imply that pamphlets are cost-ineffective. Our treatment pamphlet costs $0.07. In highly trafficked corridors, a volunteer can hand out 100 or more pamphlets in an hour. As an example, consider an opportunity cost of $15/h for a volunteer who can hand out 100 pamphlets in an hour. An effect of a 1 percentage-point decrease in meat consumption for 1 month would be equivalent to turning two average meat eaters (who eat about half of their meals with meat) into vegetarians for a month, for a total cost of $22 ($15 for the hour of pamphleteering plus $7 for the cost of 100 pamphlets). If we consider only lunch and dinner (120 meals over 1 month for two individuals), converting half of those meals from meat to vegetarian/vegan would come at cost of $0.37 per meal ($22/60 meals = $0.37). Depending on the estimated ethical (and environmental) externalities, the pamphlet could be cost effective. In contrast, a $0.37 subsidy may not be as effective at inducing a person to switch their meal from meat to vegetarian, though this an open empirical question.

We can compare the effectiveness of pamphleteering to other interventions. Two other recent studies, using real purchase data, have examined the effects of information-based interventions to reduce meat consumption. Both occur on college campuses. Jalil et al. (2020) find that students who listen to a 50-min class lecture on climate change and health reduce their meat consumption by 6.1 percentage points in the semester of the intervention, with a 95% confidence interval of [−0.094, −0.027]. Schwitzgebel et al. (2020) find that students in a philosophy class assigned to think about the ethics of eating meat reduce their meat consumption by 6.3 percentage points for several weeks, with a 95% confidence interval of [−0.102, −0.026]. Because these confidence intervals do not overlap with those from our study, we can conclude that these other interventions have larger effects.

Why are the effects of pamphleteering smaller than the effects from the classroom interventions? The classroom interventions involved nearly an hour of lecture or discussion time in the aforementioned studies, along with required readings in Schwitzgebel et al. (2020). By contrast, reading the pamphlet only takes 5–15 min, a fraction of the time of the classroom interventions, and does not involve multimedia (e.g., videos). Some students only skimmed the pamphlet and others did not read it. Another possibility is that pamphlets are less effective at challenging prior beliefs than active learning. The pamphlet addresses the same ethical issues as those in Schwitzgebel et al. (2020), but the mode of engagement, i.e., asking students to ponder and critically discuss the ethics of eating meat in a class setting, may have caused students to more directly question their preexisting notions.

In conclusion, we provide the first evidence of the effect of animal-advocacy pamphlets on meat consumption using real consumption data. Given that treatment effects are likely small, future work should focus on casting a wider net, via research designs capable of recruiting orders of magnitude more subjects. The welfare of animals on factory farms will continue to be an important issue as global demand for meat grows.

## Data Availability Statement

The datasets generated for this article are not readily available because it is owned by an anonymous college and the authors do not have permission to share it publicly. Individual participant data that underlie the results reported in this article will be made available to researchers who provide a methodologically sound proposal. Proposals should be directed to corresponding author at joshua.tasoff@cgu.edu. To gain access, data requestors will need to sign a data access agreement. Additional materials, i.e. Stata code to replicate statistical analysis, intervention materials (leaflets, survey, etc.), are publicly available.

## Ethics Statement

The studies involving human participants were reviewed and approved by the Institutional Review Board of the host college. The patients/participants provided their written informed consent to participate in this study.

## Author Contributions

MH: implementation, software, and analysis. AJ: conceptualization, experiment design, analysis, and Writing. JT: funding acquisition, conceptualization, experiment design, experiment implementation, analysis, and writing. AV: conceptualization and analysis. All authors contributed to the article and approved the submitted version.

## Conflict of Interest

The authors declare that the research was conducted in the absence of any commercial or financial relationships that could be construed as a potential conflict of interest.

## Footnotes

^1^Substituting beef for poultry and fish may have perverse ethical consequences. Because cows are large, whereas chickens and most fish are small, this substitution results in more animals slaughtered. Furthermore, the conditions under which chickens are raised are arguably worse than the conditions for cows. Both factors bring into question whether a shift from beef to poultry and fish is an ethical improvement. Due to this concern, the developers of our treatment pamphlet included three times more images of chickens and fish compared to cows (12 vs. 4).

## References

[B1] Anderson M. L. (2008). Multiple inference and gender differences in the effects of early intervention: a reevaluation of the abecedarian, perry preschool, and early training projects. J. Am. Stat. Assoc. 103, 1481–1495. 10.1198/016214508000000841

[B2] Animal Charity Evaluators (2013). Leafleting Study. Available online at: https://animalcharityevaluators.org/advocacy-interventions/interventions/leafleting/2014-march/2013-leafleting-study/ (accessed November 6, 2020).

[B3] Animal Charity Evaluators (2017). Leafleting. Available online at: https://animalcharityevaluators.org/advocacy-interventions/interventions/leafleting/ (accessed November 5, 2020).

[B4] Anthis J. R. (2017). Survey of US Attitudes Towards Animal Farming and Animal-Free Food. Available online at: https://sentienceinstitute.org/animal-farming-attitudes-survey-2017. Sentience Institute (accessed November 5, 2020).

[B5] Anthis J. R. (2019). US Factory Farming Estimates. Available online at: https://sentienceinstitute.org/us-factory-farming-estimates (accessed November 5, 2020).

[B6] Bastian B. Loughnan S. Haslam N. Radke H. R. (2012). Don't mind meat? the denial of mind to animals used for human consumption. Pers. Soc. Psychol. Bull. 38, 247–256. 10.1177/0146167211424291 21980158

[B7] Benjamini Y. Krieger A. M. Yekutieli D. (2006). Adaptive linear step-up procedures that control the false discovery rate. Biometrika 93, 491–507. 10.1093/biomet/93.3.49125152043

[B8] Bianchi F. Dorsel C. Garnett E. Aveyard P. Jebb S. A. (2018). Interventions targeting conscious determinants of human behaviour to reduce the demand for meat: a systematic review with qualitative comparative analysis. Int. J. Behav. Nutr. Phys. Act. 15, 1–25. 10.1186/s12966-018-0729-6 30340498 PMC6194670

[B9] Broom D. M. Johnson K. G. (1993). Stress and Animal Welfare, Vol. 993. Dordrecht: Springer.

[B10] Broom D. M. Mendl M. T. Zanella A. J. (1995). A comparison of the welfare of sows in different housing conditions. Ani. Sci. 61, 369–385. 10.1017/S1357729800013928

[B11] Carden L. Wood W. (2018). Habit formation and change. Curr. Opin. Behav. Sci. 20, 117–122. 10.1016/j.cobeha.2017.12.009

[B12] Cheng H. (2006). Morphopathological changes and pain in beak trimmed laying hens. Worlds Poult. Sci. J. 62, 41–52. 10.1079/WPS200583

[B13] Duncan I. J. H. (2001). Animal welfare issues in the poultry industry: is there a lesson to be learned? J. Appl. Ani. Welfare Sci. 4, 207–221. 10.1207/S15327604JAWS0403_04

[B14] Flens G. Moleman P. de Rooy L. (2018). The Effectiveness of Leafletting on Reducing the Consumption of Animal Products in Dutch Students. Working Paper.

[B15] Forman J. J. (2017). Locking Up Our Own: Crime and Punishment in Black America. New York, NY: Farrar, Straus and Giroux.

[B16] Fraser D. Mench J. Millman S. (2001). Farm Animals and Their Welfare in 2000. (Washington, DC: Humane Society Press), 87–99.

[B17] Garnett E. E. Balmford A. Sandbrook C. Pilling M. A. Marteau T. M. (2019). Impact of increasing vegetarian availability on meal selection and sales in cafeterias. Proc. Natl. Acad. Sci. U.S.A. 116, 20923–20929. 10.1073/pnas.1907207116 31570584 PMC6800350

[B18] Garnett E. E. Marteau T. M. Sandbrook C. Pilling M. A. Balmford A. (2020). Order of meals at the counter and distance between options affect student cafeteria vegetarian sales. Nat. Food 1, 485–488. 10.1038/s43016-020-0132-8 37128072

[B19] Gregory N. G. Wilkins L. J. (1989). Broken bones in domestic fowl: handling and processing damage in end-of-lay battery hens. Br. Poult. Sci. 30, 555–562. 10.1080/00071668908417179 2819499

[B20] Hansen P. G. Schilling M. Malthesen M. S. (2019). Nudging healthy and sustainable food choices: three randomized controlled field experiments using a vegetarian lunch-default as a normative signal. J. Public Health. fdz154. 10.1093/pubmed/fdz154 PMC8185453 31786590

[B21] Hennessy S. R. (2016). The Impact of Information on Animal Product Consumption. Master's thesis, University of Illinois at Urbana-Champaign.

[B22] Jalil A. J. Tasoff J. Vargas Bustamante A. (2020). Eating to save the planet: Evidence from a randomized controlled trial using individual-level food purchase data. Food Policy 95:101950. 10.1016/j.foodpol.2020.101950

[B23] Kowal J. F. (2015). The Improbable Victory of Marriage Equality. New York, NY: Brennan Center for Justice. (accessed November 5, 2020).

[B24] Marchant J. Broom D. (1996). Effects of dry sow housing conditions on muscle weight and bone strength. Animal Sci. 62, 105–114. 10.1017/S1357729800014387

[B25] Norwood B. Murray S. (2018). Food Demand Survey. Oklahoma State University, 5.

[B26] Norwood F. B. Lusk J. L. (2011). Compassion, by the Pound: The Economics of Farm Animal Welfare. Oxford: Oxford University Press.

[B27] Peacock J. Sethu H. (2017). Which Request Creates the Most Diet Change?: A Reanalysis. Technical report. Open Science Framework.

[B28] Prochaska J. O. DiClemente C. C. (1982). Transtheoretical therapy: toward a more integrative model of change. Psychotherapy 19, 276–288. 10.1037/h0088437

[B29] Prochaska J. O. DiClemente C. C. (1983). Stages and processes of self-change of smoking: toward an integrative model of change. J. Consult. Clin. Psychol. 51, 390–395. 10.1037/0022-006X.51.3.390 6863699

[B30] Regan T. (1983). Animal rights, human wrongs, in Ethics and Animals, (London: Springer), 19–43.

[B31] Rothgerber H. (2020). Meat-related cognitive dissonance: a conceptual framework for understanding how meat eaters reduce negative arousal from eating animals. Appetite 146:104511. 10.1016/j.appet.2019.104511 31707073

[B32] Sanders B. (2018). Global Animal Slaughter Statistics And Charts Olympia, WA. Available online at: https://faunalytics.org/global-animal-slaughter-statistics-and-charts/ (accessed November 5, 2020).

[B33] Schröder M. J. McEachern M. G. (2004). Consumer value conflicts surrounding ethical food purchase decisions: a focus on animal welfare. Int. J. Cons. Stud. 28, 168–177. 10.1111/j.1470-6431.2003.00357.x

[B34] Schwitzgebel E. Cokelet B. Singer P. (2020). Do ethics classes influence student behavior? Case study: teaching the ethics of eating meat. Cognition 203:104397. 10.1016/j.cognition.2020.104397 32721655

[B35] Singer P. (1979). Killing humans and Killing animals. Inquiry 22, 145–156. 10.1080/00201747908601869

[B36] Sonoda Y. Oishi K. Chomei Y. Hirooka H. (2018). How do human values influence the beef preferences of consumer segments regarding animal welfare and environmentally friendly production? Meat Sci. 146, 75–86. 10.1016/j.meatsci.2018.07.030 30103081

[B37] Vandenbroele J. Slabbinck H. Van Kerckhove A. Vermeir I. (2019). Mock meat in the butchery: nudging consumers toward meat substitutes. Organ. Behav. Hum. Decis. Proc. 163, 105–116. 10.1016/j.obhdp.2019.09.004

[B38] Vieuille-Thomas C. Pape G. L. Signoret J. P. (1995). Stereotypies in pregnant sows: indications of influence of the housing system on the patterns expressed by the animals. Appl. Ani. Behav. Sci. 44, 19–27. 10.1016/0168-1591(95)00574-C

