# Supplementary material for: Changing Hearts and Plates: The Effect of Animal-Advocacy Pamphlets on Meat Consumption
Source: Front Psychol. 2021 May 31;12:668674. doi: 10.3389/fpsyg.2021.668674 (PMC8220289; doi:10.3389/fpsyg.2021.668674)
Supplement: Supplementary file 1 [file Presentation_1.pdf]

# Supplemental Information

May 10, 2021

# Contents

**1 Additional Results** **4**
1.1 Balance . . . 4
1.2 Effect of the Treatment on the Survey . . . 5
1.3 Additional Heterogeneous Treatment Effects . . . 6

**2 Experiment Materials** **21**
2.1 Pamphlets . . . 21
2.2 Qualtrics Survey . . . 40

**3 Pre-Analysis Plan (PAP)** **53**

# List of Tables


A.1 Balance Table by Treatment . . . . . 4
A.2 Selection into Survey. Treatment Condition Only . . . . . 4
A.3 Selection into Survey. Control Condition Only . . . . . 5
A.4 Balance Table of Survey Questions by Treatment . . . . . 6
A.5 Hetero effect reducer: first month, second month, after 2 months . . . . . 7
A.6 Hetero effect diet changed: first month, second month, after 2 months . . . . . 8
A.7 Hetero effect identify correct leaflet: first month, second month, after 2 months . . . 9
A.8 Hetero effect read leaflet: first month, second month, after 2 months . . . . . 10
A.9 Hetero effect leaflet affected me I am eating different: first month, second month, after 2 months . . . . . 11
A.10 Mechanism taught me: first month, second month, after 2 months . . . . . 12
A.11 Mechanism thought more: first month, second month, after 2 months . . . . . 13
A.12 Mechanism willingness: first month, second month, after 2 months . . . . . 14
A.13 Hetero effect treatment of animals is extremely important: first month, second month, after 2 months . . . . . 15
A.14 Hetero effect personal action is extremely impactful : first month, second month, after 2 months . . . . . 16
A.15 Hetero effect plant based meals are easily accessible: first month, second month, after 2 months . . . . . 17
A.16 Hetero effect survey taker: first month, second month, after 2 months . . . . . 18
A.17 Hetero effect survey taker and gender: first month, second month, after 2 months . 19
A.18 Main result: including post . . . . . 20

# 1 Additional Results

## 1.1 Balance

Our treatment was assigned randomly. Therefore, in expectation, covariates should be balanced across the two conditions. We test for balance below. We find no differences in pre-intervention meat consumption across conditions.

Table A.1: Balance Table by Treatment

| | Means | | Difference |
|---|---|---|---|
| Variable | Pre-Intervention Contr. | Pre-Intervention Treat. | Treat. − Contr. |
| beef | 0.162 | 0.160 | -0.002 |
| | (0.368) | (0.366) | (0.826) |
| poultryfish | 0.394 | 0.397 | 0.003 |
| | (0.489) | (0.489) | (0.805) |
| meat | 0.556 | 0.557 | 0.001 |
| | (0.497) | (0.497) | (0.938) |
| veg | 0.280 | 0.268 | -0.012 |
| | (0.449) | (0.443) | (0.403) |
| Observations | 42,725 | 43,846 | 86,571 |

The survey was not randomly assigned. There may be selection into the survey. We find that in both the treatment and control conditions, survey-takers eat less meat than those who attrit.

Table A.2: Selection into Survey. Treatment Condition Only

| | Means | | Difference |
|---|---|---|---|
| Variable | Survey=1 | Survey=0 | took survey − didn't take survey |
| beef | 0.145 | 0.173 | -0.027** |
| | (0.353) | (0.378) | (0.016) |
| poultryfish | 0.378 | 0.413 | -0.035* |
| | (0.485) | (0.492) | (0.066) |
| meat | 0.524 | 0.586 | -0.062** |
| | (0.499) | (0.493) | (0.013) |
| veg | 0.300 | 0.247 | 0.053*** |
| | (0.458) | (0.431) | (0.006) |
| Observations | 48,203 | 53,347 | 101,550 |

Table A.3: Selection into Survey. Control Condition Only

| | Means | | Difference |
|---|---|---|---|
| Variable | Survey=1 | Survey=0 | took survey − didn't take survey |
| beef | 0.157 | 0.169 | -0.011 |
| | (0.364) | (0.374) | (0.374) |
| poultryfish | 0.371 | 0.413 | -0.042** |
| | (0.483) | (0.492) | (0.020) |
| meat | 0.528 | 0.582 | -0.053** |
| | (0.499) | (0.493) | (0.037) |
| veg | 0.314 | 0.259 | 0.055*** |
| | (0.464) | (0.438) | (0.005) |
| Observations | 49,128 | 49,365 | 98,493 |

## 1.2 Effect of the Treatment on the Survey

The treatment condition may affect the survey responses. Table A.4 shows the effect of the treatment on the individual survey questions. The treatment significantly increased the probability of identifying the correct leaflet received, increased the probability of "affected me eating and thinking differently", increased the probability of being "taught about treatment of animals in farms", and increased the probability that the person "thought more about treatment of animals in farms".

Table A.4: Balance Table of Survey Questions by Treatment

| | Means | | Difference |
|---|---|---|---|
| Variable | Control | Treatment | Treat. − Contr. |
| vegan/vegetarian diet | 0.178 | 0.121 | -0.057 |
| | (0.383) | (0.326) | (0.254) |
| meat reduction diet | 0.210 | 0.157 | -0.052 |
| | (0.407) | (0.364) | (0.309) |
| has changed diet | 0.141 | 0.131 | -0.010 |
| | (0.348) | (0.337) | (0.827) |
| Reason dietchanged:Animal cruelty and ethics | 0.081 | 0.026 | -0.056 |
| | (0.274) | (0.159) | (0.496) |
| identified the correct leaflet | 0.468 | 0.723 | 0.256*** |
| | (0.499) | (0.447) | (0.000) |
| read all, some, glaced at the leaflet | 0.838 | 0.913 | 0.075 |
| | (0.369) | (0.282) | (0.149) |
| leaflet has affected me eating and thinking differently | 0.213 | 0.447 | 0.233*** |
| | (0.410) | (0.497) | (0.001) |
| the leaflet taught me about treatment of animals in farms | 0.126 | 0.600 | 0.474*** |
| | (0.332) | (0.490) | (0.000) |
| I thought more about treatment of animals in farms | 0.239 | 0.615 | 0.377*** |
| | (0.426) | (0.487) | (0.000) |
| animal treatment extremely important | 0.522 | 0.476 | -0.045 |
| | (0.500) | (0.499) | (0.507) |
| personal action extremely impactful | 0.252 | 0.213 | -0.039 |
| | (0.434) | (0.410) | (0.506) |
| animal product replacement easily accessible | 0.212 | 0.259 | 0.047 |
| | (0.409) | (0.438) | (0.398) |
| willingess to make lifestyle changes to reduce mistreatment of farm animals | 1.663 | 1.579 | -0.084 |
| | (0.676) | (0.655) | (0.370) |
| Observations | 9,012 | 9,227 | 18,239 |

## 1.3 Additional Heterogeneous Treatment Effects

This section shows the results from additional analyses that test for heterogenous treatment effects.

Table A.5: Hetero effect reducer: first month, second month, after 2 months

| | first month | | | | second month | | | | after 2 months | | | |
|---|---|---|---|---|---|---|---|---|---|---|---|---|
| | (1) | (2) | (3) | (4) | (5) | (6) | (7) | (8) | (9) | (10) | (11) | (12) |
| | beef | poultry/fish | veg | meat | beef | poultry/fish | veg | meat | beef | poultry/fish | veg | meat |
| treated | -0.011 | -0.002 | 0.024 | -0.011 | -0.011 | 0.010 | 0.003 | -0.001 | -0.011 | 0.000 | 0.001 | -0.011 |
| | (0.010) | (0.014) | (0.014) | (0.015) | (0.010) | (0.016) | (0.014) | (0.016) | (0.008) | (0.016) | (0.015) | (0.017) |
| treated × reducer | 0.033 | -0.008 | -0.016 | 0.017 | 0.029 | 0.008 | -0.038 | 0.032 | 0.045 | 0.018 | -0.012 | 0.051 |
| | (0.027) | (0.034) | (0.035) | (0.033) | (0.032) | (0.037) | (0.034) | (0.038) | (0.032) | (0.037) | (0.038) | (0.035) |
| P-value reducer | 0.359 | 0.758 | 0.803 | 0.847 | 0.538 | 0.580 | 0.263 | 0.355 | 0.253 | 0.574 | 0.759 | 0.193 |
| Mean of untreated DV reducer=0 | .153 | .369 | .306 | .522 | .15 | .373 | .304 | .522 | .155 | .37 | .305 | .525 |
| Mean of untreated DV reducer=1 | .139 | .398 | .28 | .537 | .136 | .403 | .277 | .539 | .143 | .391 | .297 | .534 |
| PseudoR2 | .13 | .124 | .154 | .167 | .131 | .121 | .154 | .166 | .126 | .117 | .154 | .166 |
| Clusters | 336 | 342 | 342 | 343 | 335 | 342 | 342 | 342 | 339 | 342 | 342 | 343 |
| N | 51,830 | 52,174 | 52,203 | 52,237 | 51,233 | 51,540 | 51,581 | 51,595 | 77,377 | 77,633 | 77,579 | 77,661 |

Table A.6: Hetero effect diet changed: first month, second month, after 2 months

| | first month | | | | second month | | | | after 2 months | | | |
|---|---|---|---|---|---|---|---|---|---|---|---|---|
| | (1) beef | (2) poultry/fish | (3) veg | (4) meat | (5) beef | (6) poultry/fish | (7) veg | (8) meat | (9) beef | (10) poultry/fish | (11) veg | (12) meat |
| treated | -0.003 | -0.002 | 0.016 | -0.003 | -0.005 | 0.011 | -0.004 | 0.007 | -0.010 | 0.008 | 0.007 | -0.003 |
| | (0.010) | (0.013) | (0.013) | (0.014) | (0.010) | (0.015) | (0.014) | (0.015) | (0.008) | (0.016) | (0.015) | (0.017) |
| treated × dietchanged | -0.022 | -0.002 | 0.026 | -0.025 | -0.003 | 0.004 | 0.006 | -0.007 | 0.031 | -0.027 | -0.048 | -0.000 |
| | (0.023) | (0.044) | (0.050) | (0.044) | (0.029) | (0.049) | (0.039) | (0.048) | (0.024) | (0.037) | (0.034) | (0.038) |
| P-value dietchanged | 0.243 | 0.926 | 0.394 | 0.509 | 0.765 | 0.744 | 0.958 | 0.997 | 0.355 | 0.573 | 0.193 | 0.922 |
| Mean of untreated DV dietchanged=0 | .151 | .37 | .309 | .521 | .148 | .374 | .308 | .522 | .154 | .37 | .309 | .524 |
| Mean of untreated DV dietchanged=1 | .146 | .395 | .259 | .542 | .144 | .398 | .254 | .542 | .15 | .391 | .274 | .541 |
| PseudoR2 | .13 | .124 | .154 | .167 | .132 | .121 | .154 | .167 | .126 | .117 | .154 | .166 |
| Clusters | 335 | 341 | 342 | 342 | 334 | 341 | 341 | 341 | 338 | 341 | 342 | 342 |
| N | 51,808 | 52,152 | 52,203 | 52,210 | 51,211 | 51,518 | 51,559 | 51,573 | 77,357 | 77,613 | 77,579 | 77,641 |

Table A.7: Hetero effect identify correct leaflet: first month, second month, after 2 months

| | first month | | | | second month | | | | after 2 months | | | |
|---|---|---|---|---|---|---|---|---|---|---|---|---|
| | (1) | (2) | (3) | (4) | (5) | (6) | (7) | (8) | (9) | (10) | (11) | (12) |
| | beef | poultry/fish | veg | meat | beef | poultry/fish | veg | meat | beef | poultry/fish | veg | meat |
| treated | -0.004 | 0.006 | 0.015 | 0.003 | -0.002 | 0.011 | -0.001 | 0.006 | -0.016 | 0.013 | 0.002 | -0.003 |
| | (0.014) | (0.024) | (0.027) | (0.028) | (0.015) | (0.023) | (0.020) | (0.023) | (0.013) | (0.025) | (0.024) | (0.026) |
| treated × correctleaflet | -0.004 | -0.011 | 0.009 | -0.016 | -0.005 | 0.006 | -0.000 | 0.006 | 0.015 | -0.004 | -0.004 | 0.010 |
| | (0.020) | (0.029) | (0.031) | (0.033) | (0.020) | (0.031) | (0.027) | (0.031) | (0.018) | (0.032) | (0.031) | (0.033) |
| P-value correctleaflet | 0.573 | 0.730 | 0.148 | 0.440 | 0.633 | 0.405 | 0.944 | 0.536 | 0.920 | 0.640 | 0.937 | 0.729 |
| Mean of untreated DV correctleaflet=0 | .171 | .398 | .263 | .569 | .166 | .402 | .263 | .568 | .171 | .398 | .269 | .57 |
| Mean of untreated DV correctleaflet=1 | .134 | .354 | .332 | .488 | .132 | .358 | .328 | .49 | .138 | .352 | .332 | .49 |
| PseudoR2 | .129 | .125 | .153 | .167 | .131 | .123 | .153 | .166 | .125 | .117 | .152 | .164 |
| Clusters | 328 | 334 | 334 | 335 | 327 | 334 | 334 | 334 | 331 | 334 | 334 | 335 |
| N | 50,382 | 50,725 | 50,754 | 50,788 | 49,788 | 50,105 | 50,146 | 50,160 | 75,013 | 75,297 | 75,243 | 75,325 |

Table A.8: Hetero effect read leaflet: first month, second month, after 2 months

| | first month | | | | second month | | | | after 2 months | | | |
|---|---|---|---|---|---|---|---|---|---|---|---|---|
| | (1) | (2) | (3) | (4) | (5) | (6) | (7) | (8) | (9) | (10) | (11) | (12) |
| | beef | poultry/fish | veg | meat | beef | poultry/fish | veg | meat | beef | poultry/fish | veg | meat |
| treated | 0.007 | 0.068** | -0.050 | 0.075* | -0.020 | -0.012 | 0.030 | -0.039 | -0.021 | -0.016 | -0.048 | -0.040 |
| | (0.026) | (0.034) | (0.041) | (0.042) | (0.026) | (0.048) | (0.041) | (0.052) | (0.024) | (0.040) | (0.048) | (0.037) |
| treated × readleaflet | -0.015 | -0.070** | 0.085* | -0.090* | 0.020 | 0.033 | -0.030 | 0.058 | 0.026 | 0.014 | 0.062 | 0.042 |
| | (0.027) | (0.035) | (0.051) | (0.046) | (0.035) | (0.053) | (0.042) | (0.054) | (0.030) | (0.045) | (0.056) | (0.042) |
| P-value readleaflet | 0.510 | 0.885 | 0.056 | 0.331 | 0.993 | 0.271 | 0.986 | 0.269 | 0.655 | 0.915 | 0.447 | 0.937 |
| Mean of untreated DV readleaflet=0 | .162 | .405 | .253 | .567 | .163 | .409 | .25 | .572 | .164 | .403 | .248 | .567 |
| Mean of untreated DV readleaflet=1 | .139 | .356 | .33 | .494 | .136 | .361 | .326 | .496 | .14 | .355 | .331 | .495 |
| PseudoR2 | .124 | .137 | .166 | .186 | .125 | .136 | .166 | .185 | .121 | .13 | .164 | .183 |
| Clusters | 233 | 237 | 238 | 238 | 232 | 237 | 237 | 237 | 236 | 237 | 238 | 238 |
| N | 35,647 | 36,009 | 36,048 | 36,069 | 35,374 | 35,660 | 35,690 | 35,711 | 52,886 | 53,096 | 53,058 | 53,125 |

Table A.9: Hetero effect leaflet affected me I am eating different: first month, second month, after 2 months

| | first month | | | | second month | | | | after 2 months | | | |
|---|---|---|---|---|---|---|---|---|---|---|---|---|
| | (1) beef | (2) poultry/fish | (3) veg | (4) meat | (5) beef | (6) poultry/fish | (7) veg | (8) meat | (9) beef | (10) poultry/fish | (11) veg | (12) meat |
| treated | -0.009 | 0.018 | 0.012 | 0.010 | 0.007 | 0.011 | -0.006 | 0.018 | 0.001 | -0.003 | -0.001 | -0.001 |
| | (0.014) | (0.017) | (0.017) | (0.018) | (0.014) | (0.022) | (0.018) | (0.022) | (0.013) | (0.021) | (0.022) | (0.022) |
| treated × affectedme | 0.019 | -0.023 | 0.005 | -0.007 | -0.021 | 0.001 | 0.008 | -0.016 | 0.001 | -0.042 | 0.051 | -0.044 |
| | (0.027) | (0.032) | (0.032) | (0.030) | (0.021) | (0.032) | (0.033) | (0.034) | (0.019) | (0.033) | (0.038) | (0.035) |
| P-value affectedme | 0.670 | 0.845 | 0.531 | 0.895 | 0.389 | 0.621 | 0.926 | 0.948 | 0.872 | 0.085 | 0.097 | 0.108 |
| Mean of untreated DV affectedme=0 | .132 | .351 | .339 | .483 | .131 | .354 | .335 | .485 | .134 | .34 | .346 | .474 |
| Mean of untreated DV affectedme=1 | .157 | .379 | .29 | .536 | .152 | .387 | .289 | .539 | .161 | .397 | .276 | .558 |
| PseudoR2 | .124 | .138 | .167 | .187 | .126 | .136 | .167 | .185 | .121 | .131 | .165 | .183 |
| Clusters | 234 | 238 | 239 | 239 | 233 | 238 | 238 | 238 | 237 | 238 | 239 | 239 |
| N | 35,792 | 36,154 | 36,193 | 36,214 | 35,497 | 35,783 | 35,813 | 35,834 | 52,992 | 53,202 | 53,164 | 53,231 |

Table A.10: Mechanism taught me: first month, second month, after 2 months

| | first month | | | | second month | | | | after 2 months | | | |
|---|---|---|---|---|---|---|---|---|---|---|---|---|
| | (1) | (2) | (3) | (4) | (5) | (6) | (7) | (8) | (9) | (10) | (11) | (12) |
| | beef | poultry/fish | veg | meat | beef | poultry/fish | veg | meat | beef | poultry/fish | veg | meat |
| treated | -0.018 | -0.021 | 0.037** | -0.036** | 0.003 | 0.004 | 0.010 | 0.010 | -0.009 | 0.007 | 0.025 | -0.001 |
| | (0.014) | (0.019) | (0.018) | (0.017) | (0.016) | (0.020) | (0.021) | (0.021) | (0.015) | (0.019) | (0.023) | (0.024) |
| treated × taughtme | 0.041 | 0.017 | -0.030 | 0.051 | -0.019 | -0.010 | -0.019 | -0.026 | 0.083** | -0.093** | -0.005 | -0.026 |
| | (0.032) | (0.035) | (0.039) | (0.033) | (0.029) | (0.032) | (0.041) | (0.031) | (0.042) | (0.039) | (0.043) | (0.041) |
| P-value taughtme | 0.376 | 0.891 | 0.845 | 0.602 | 0.525 | 0.814 | 0.815 | 0.471 | 0.053 | 0.017 | 0.609 | 0.441 |
| Mean of untreated DV taughtme=0 | .143 | .356 | .325 | .499 | .14 | .362 | .32 | .502 | .145 | .352 | .327 | .497 |
| Mean of untreated DV taughtme=1 | .15 | .373 | .312 | .524 | .147 | .379 | .31 | .527 | .152 | .378 | .31 | .53 |
| PseudoR2 | .127 | .144 | .173 | .196 | .127 | .143 | .174 | .195 | .121 | .135 | .172 | .188 |
| Clusters | 205 | 208 | 209 | 209 | 204 | 208 | 208 | 208 | 207 | 208 | 209 | 209 |
| N | 31,634 | 31,855 | 31,902 | 31,922 | 31,601 | 31,778 | 31,821 | 31,841 | 47,140 | 47,345 | 47,321 | 47,386 |

Table A.11: Mechanism thought more: first month, second month, after 2 months

| | first month | | | | second month | | | | after 2 months | | | |
|---|---|---|---|---|---|---|---|---|---|---|---|---|
| | (1) | (2) | (3) | (4) | (5) | (6) | (7) | (8) | (9) | (10) | (11) | (12) |
| | beef | poultry/fish | veg | meat | beef | poultry/fish | veg | meat | beef | poultry/fish | veg | meat |
| treated | -0.006 | -0.011 | $0.049^{**}$ | -0.015 | 0.007 | $0.035^{*}$ | -0.008 | $0.042^{*}$ | -0.009 | 0.022 | -0.012 | 0.012 |
| | (0.016) | (0.019) | (0.019) | (0.018) | (0.018) | (0.021) | (0.021) | (0.022) | (0.014) | (0.024) | (0.024) | (0.026) |
| treated × thoughtmore | -0.010 | 0.000 | -0.007 | -0.013 | -0.016 | $-0.071^{**}$ | 0.040 | $-0.084^{***}$ | 0.022 | $-0.080^{**}$ | $0.071^{*}$ | -0.061 |
| | (0.023) | (0.031) | (0.030) | (0.030) | (0.025) | (0.029) | (0.033) | (0.030) | (0.024) | (0.033) | (0.043) | (0.042) |
| P-value thoughtmore | 0.350 | 0.661 | 0.095 | 0.250 | 0.619 | 0.126 | 0.192 | 0.034 | 0.484 | 0.017 | 0.092 | 0.135 |
| Mean of untreated DV thoughtmore=0 | .156 | .374 | .303 | .53 | .153 | .378 | .299 | .531 | .156 | .365 | .306 | .522 |
| Mean of untreated DV thoughtmore=1 | .132 | .354 | .333 | .486 | .129 | .36 | .331 | .489 | .137 | .361 | .332 | .498 |
| PseudoR2 | .127 | .141 | .172 | .192 | .127 | .14 | .173 | .191 | .121 | .133 | .172 | .185 |
| Clusters | 210 | 213 | 214 | 214 | 209 | 213 | 213 | 213 | 212 | 213 | 214 | 214 |
| N | 32,573 | 32,797 | 32,840 | 32,860 | 32,504 | 32,684 | 32,723 | 32,743 | 48,214 | 48,419 | 48,391 | 48,456 |

Table A.12: Mechanism willingness: first month, second month, after 2 months

| | first month | | | | second month | | | | after 2 months | | | |
|---|---|---|---|---|---|---|---|---|---|---|---|---|
| | (1) beef | (2) poultry/fish | (3) veg | (4) meat | (5) beef | (6) poultry/fish | (7) veg | (8) meat | (9) beef | (10) poultry/fish | (11) veg | (12) meat |
| treated | 0.044 | -0.054 | 0.105 | -0.053 | 0.052* | 0.076 | -0.041 | 0.120 | -0.028 | 0.134*** | -0.013 | 0.075* |
| | (0.039) | (0.078) | (0.089) | (0.120) | (0.029) | (0.093) | (0.065) | (0.089) | (0.040) | (0.052) | (0.033) | (0.039) |
| treated × smallchange | -0.041 | 0.070 | -0.091 | 0.066 | -0.054*** | -0.058 | 0.034 | -0.124 | 0.016 | -0.130*** | 0.025 | -0.094** |
| | (0.030) | (0.086) | (0.068) | (0.116) | (0.021) | (0.089) | (0.077) | (0.099) | (0.046) | (0.049) | (0.042) | (0.047) |
| treated × moderatechange | -0.046 | 0.047 | -0.065 | 0.035 | -0.044* | -0.058 | 0.040 | -0.113 | 0.029 | -0.110** | -0.005 | -0.058 |
| | (0.029) | (0.086) | (0.072) | (0.119) | (0.024) | (0.088) | (0.076) | (0.098) | (0.049) | (0.051) | (0.040) | (0.045) |
| treated × bigchange | -0.043 | -0.123 | -0.051 | -0.075 | -0.041 | -0.200** | 0.090 | -0.232** | 0.088 | -0.246** | 0.049 | -0.155 |
| | (0.045) | (0.150) | (0.084) | (0.167) | (0.059) | (0.097) | (0.089) | (0.099) | (0.071) | (0.099) | (0.060) | (0.113) |
| P-value smallchange | 0.864 | 0.377 | 0.610 | 0.437 | 0.862 | 0.413 | 0.783 | 0.865 | 0.329 | 0.822 | 0.626 | 0.452 |
| P-value moderatechange | 0.932 | 0.746 | 0.119 | 0.359 | 0.629 | 0.404 | 0.961 | 0.739 | 0.908 | 0.223 | 0.382 | 0.434 |
| P-value bigchange | 0.985 | 0.186 | 0.248 | 0.273 | 0.853 | 0.119 | 0.269 | 0.027 | 0.211 | 0.242 | 0.473 | 0.462 |
| Mean of untreated DV nochange | .226 | .357 | .24 | .584 | .214 | .369 | .242 | .583 | .228 | .359 | .228 | .587 |
| Mean of untreated DV smallchange | .196 | .442 | .225 | .638 | .192 | .446 | .223 | .638 | .196 | .437 | .231 | .633 |
| Mean of untreated DV moderatechange | .128 | .36 | .327 | .488 | .125 | .362 | .326 | .487 | .131 | .363 | .328 | .494 |
| Mean of untreated DV bigchange | .0467 | .102 | .568 | .148 | .0462 | .112 | .554 | .158 | .047 | .116 | .553 | .163 |
| PseudoR2 | .13 | .126 | .154 | .169 | .131 | .123 | .154 | .168 | .125 | .118 | .153 | .166 |
| Clusters | 327 | 333 | 333 | 334 | 326 | 333 | 333 | 333 | 330 | 333 | 333 | 334 |
| N | 50,288 | 50,631 | 50,660 | 50,694 | 49,749 | 50,066 | 50,107 | 50,121 | 75,193 | 75,477 | 75,423 | 75,505 |

Table A.13: Hetero effect treatment of animals is extremely important: first month, second month, after 2 months

| | first month | | | | second month | | | | after 2 months | | | |
|---|---|---|---|---|---|---|---|---|---|---|---|---|
| | (1) | (2) | (3) | (4) | (5) | (6) | (7) | (8) | (9) | (10) | (11) | (12) |
| | beef | poultry/fish | veg | meat | beef | poultry/fish | veg | meat | beef | poultry/fish | veg | meat |
| treated | -0.018 | -0.014 | 0.027 | -0.034* | -0.000 | -0.017 | 0.007 | -0.020 | -0.009 | -0.003 | -0.003 | -0.013 |
| | (0.011) | (0.018) | (0.020) | (0.019) | (0.012) | (0.018) | (0.018) | (0.019) | (0.010) | (0.018) | (0.018) | (0.021) |
| treated × extremelyimportant | 0.034 | 0.030 | -0.012 | 0.060** | -0.015 | 0.068** | -0.019 | 0.057** | 0.009 | 0.022 | -0.001 | 0.031 |
| | (0.022) | (0.027) | (0.025) | (0.027) | (0.018) | (0.030) | (0.026) | (0.029) | (0.017) | (0.030) | (0.029) | (0.031) |
| P-value extremelyimportant | 0.363 | 0.435 | 0.392 | 0.193 | 0.284 | 0.029 | 0.540 | 0.103 | 0.977 | 0.452 | 0.877 | 0.431 |
| Mean of untreated DV extremelyimportant=0 | .171 | .405 | .273 | .576 | .167 | .41 | .272 | .577 | .176 | .409 | .268 | .586 |
| Mean of untreated DV extremelyimportant=1 | .128 | .334 | .339 | .462 | .125 | .337 | .335 | .462 | .128 | .331 | .346 | .459 |
| PseudoR2 | .129 | .125 | .154 | .168 | .131 | .123 | .154 | .168 | .125 | .117 | .153 | .166 |
| Clusters | 327 | 333 | 333 | 334 | 326 | 333 | 333 | 333 | 330 | 333 | 333 | 334 |
| N | 50,288 | 50,631 | 50,660 | 50,694 | 49,749 | 50,066 | 50,107 | 50,121 | 75,193 | 75,477 | 75,423 | 75,505 |

Table A.14: Hetero effect personal action is extremely impactful : first month, second month, after 2 months

| | first month | | | | second month | | | | after 2 months | | | |
|---|---|---|---|---|---|---|---|---|---|---|---|---|
| | (1) beef | (2) poultry/fish | (3) veg | (4) meat | (5) beef | (6) poultry/fish | (7) veg | (8) meat | (9) beef | (10) poultry/fish | (11) veg | (12) meat |
| treated | -0.011 | -0.001 | 0.026* | -0.010 | -0.003 | -0.005 | 0.014 | -0.008 | -0.006 | -0.012 | 0.007 | -0.018 |
| | (0.009) | (0.014) | (0.015) | (0.015) | (0.010) | (0.016) | (0.014) | (0.016) | (0.009) | (0.015) | (0.016) | (0.016) |
| treated × extremelyimpactful | 0.024 | -0.001 | -0.016 | 0.017 | -0.026 | 0.088** | -0.058* | 0.059 | -0.005 | 0.095** | -0.028 | 0.080** |
| | (0.033) | (0.040) | (0.033) | (0.040) | (0.030) | (0.044) | (0.031) | (0.042) | (0.019) | (0.047) | (0.034) | (0.040) |
| P-value extremelyimpactful | 0.678 | 0.968 | 0.731 | 0.854 | 0.311 | 0.044 | 0.126 | 0.181 | 0.516 | 0.062 | 0.484 | 0.090 |
| Mean of untreated DV extremelyimpactful=0 | .16 | .386 | .289 | .545 | .156 | .391 | .287 | .547 | .166 | .391 | .284 | .556 |
| Mean of untreated DV extremelyimpactful=1 | .119 | .324 | .354 | .443 | .117 | .323 | .353 | .441 | .106 | .302 | .383 | .408 |
| PseudoR2 | .129 | .125 | .154 | .168 | .131 | .123 | .154 | .167 | .125 | .118 | .153 | .167 |
| Clusters | 327 | 333 | 333 | 334 | 326 | 333 | 333 | 333 | 330 | 333 | 333 | 334 |
| N | 50,288 | 50,631 | 50,660 | 50,694 | 49,749 | 50,066 | 50,107 | 50,121 | 75,193 | 75,477 | 75,423 | 75,505 |

Table A.15: Hetero effect plant based meals are easily accessible: first month, second month, after 2 months

| | first month | | | | second month | | | | after 2 months | | | |
|---|---|---|---|---|---|---|---|---|---|---|---|---|
| | (1) beef | (2) poultry/fish | (3) veg | (4) meat | (5) beef | (6) poultry/fish | (7) veg | (8) meat | (9) beef | (10) poultry/fish | (11) veg | (12) meat |
| treated | -0.004 | -0.002 | 0.019 | -0.004 | -0.000 | 0.018 | -0.003 | 0.018 | -0.013 | 0.007 | 0.006 | -0.006 |
| | (0.011) | (0.016) | (0.016) | (0.017) | (0.011) | (0.018) | (0.016) | (0.018) | (0.009) | (0.016) | (0.016) | (0.018) |
| treated × easilyaccessible | -0.011 | 0.001 | 0.006 | -0.010 | -0.028 | -0.023 | 0.003 | -0.046 | 0.045** | -0.001 | -0.031 | 0.035 |
| | (0.019) | (0.031) | (0.028) | (0.029) | (0.020) | (0.031) | (0.028) | (0.032) | (0.022) | (0.037) | (0.034) | (0.036) |
| P-value easilyaccessible | 0.360 | 0.973 | 0.281 | 0.577 | 0.104 | 0.851 | 0.984 | 0.280 | 0.102 | 0.859 | 0.397 | 0.359 |
| Mean of untreated DV easilyaccessible=0 | .162 | .397 | .279 | .559 | .157 | .401 | .278 | .559 | .164 | .398 | .279 | .562 |
| Mean of untreated DV easilyaccessible=1 | .119 | .3 | .374 | .419 | .119 | .302 | .369 | .421 | .119 | .293 | .386 | .413 |
| PseudoR2 | .129 | .125 | .154 | .168 | .131 | .123 | .154 | .167 | .125 | .117 | .153 | .166 |
| Clusters | 327 | 333 | 333 | 334 | 326 | 333 | 333 | 333 | 330 | 333 | 333 | 334 |
| N | 50,288 | 50,631 | 50,660 | 50,694 | 49,749 | 50,066 | 50,107 | 50,121 | 75,193 | 75,477 | 75,423 | 75,505 |

Table A.16: Hetero effect survey taker: first month, second month, after 2 months

| | first month | | | | second month | | | | after 2 months | | | |
|---|---|---|---|---|---|---|---|---|---|---|---|---|
| | (1) beef | (2) poultry/fish | (3) veg | (4) meat | (5) beef | (6) poultry/fish | (7) veg | (8) meat | (9) beef | (10) poultry/fish | (11) veg | (12) meat |
| treated | -0.007 | -0.006 | -0.001 | -0.012 | 0.002 | -0.014 | 0.012 | -0.014 | 0.001 | 0.002 | 0.002 | 0.004 |
| | (0.009) | (0.012) | (0.011) | (0.012) | (0.009) | (0.012) | (0.013) | (0.013) | (0.009) | (0.012) | (0.013) | (0.013) |
| treated × survey | -0.000 | 0.003 | 0.021 | 0.003 | -0.010 | 0.025 | -0.014 | 0.018 | -0.008 | 0.002 | -0.003 | -0.006 |
| | (0.013) | (0.018) | (0.018) | (0.018) | (0.013) | (0.019) | (0.018) | (0.019) | (0.012) | (0.019) | (0.019) | (0.021) |
| P-value survey | 0.486 | 0.798 | 0.129 | 0.512 | 0.448 | 0.458 | 0.820 | 0.793 | 0.419 | 0.792 | 0.937 | 0.872 |
| Mean of untreated DV survey=0 | .171 | .413 | .255 | .584 | .168 | .418 | .253 | .586 | .173 | .412 | .25 | .585 |
| Mean of untreated DV survey=1 | .15 | .374 | .302 | .524 | .147 | .377 | .3 | .525 | .153 | .373 | .304 | .527 |
| PseudoR2 | .121 | .109 | .138 | .154 | .12 | .108 | .135 | .154 | .115 | .102 | .134 | .15 |
| Clusters | 675 | 685 | 685 | 685 | 671 | 685 | 685 | 684 | 677 | 685 | 685 | 685 |
| N | 106,331 | 106,847 | 106,970 | 106,892 | 104,071 | 104,629 | 104,757 | 104,677 | 159,585 | 159,856 | 159,947 | 159,869 |

Table A.17: Hetero effect survey taker and gender: first month, second month, after 2 months

| | first month | | | | second month | | | | after 2 months | | | |
|---|---|---|---|---|---|---|---|---|---|---|---|---|
| | (1) | (2) | (3) | (4) | (5) | (6) | (7) | (8) | (9) | (10) | (11) | (12) |
| | beef | poultry/fish | veg | meat | beef | poultry/fish | veg | meat | beef | poultry/fish | veg | meat |
| treated | 0.001 | -0.021 | 0.017 | -0.024 | 0.026** | -0.044*** | 0.018 | -0.021 | 0.008 | -0.015 | 0.018 | -0.005 |
| | (0.011) | (0.016) | (0.016) | (0.018) | (0.012) | (0.016) | (0.017) | (0.017) | (0.011) | (0.019) | (0.021) | (0.022) |
| treated × survey | -0.013 | 0.010 | 0.017 | -0.003 | -0.026 | 0.044 | 0.002 | 0.018 | -0.024 | -0.002 | -0.001 | -0.038 |
| | (0.018) | (0.030) | (0.031) | (0.033) | (0.016) | (0.029) | (0.027) | (0.029) | (0.015) | (0.030) | (0.028) | (0.034) |
| treated × female | -0.016 | 0.031 | -0.031 | 0.022 | -0.051*** | 0.062** | -0.011 | 0.012 | -0.015 | 0.034 | -0.026 | 0.017 |
| | (0.016) | (0.024) | (0.021) | (0.024) | (0.015) | (0.025) | (0.025) | (0.025) | (0.016) | (0.025) | (0.025) | (0.027) |
| treated × survey × female | 0.028 | -0.019 | 0.013 | 0.004 | 0.054 | -0.043 | -0.021 | -0.002 | 0.037 | -0.000 | 0.001 | 0.043 |
| | (0.030) | (0.037) | (0.037) | (0.040) | (0.034) | (0.037) | (0.035) | (0.039) | (0.027) | (0.039) | (0.037) | (0.041) |
| P-value survey × female | 0.425 | 0.384 | 0.526 | 0.690 | 0.043 | 0.061 | 0.948 | 0.640 | 0.186 | 0.764 | 0.717 | 0.499 |
| Mean of untreated DV survey=0 | .171 | .413 | .255 | .584 | .168 | .418 | .253 | .586 | .173 | .412 | .25 | .585 |
| Mean of untreated DV survey=1 | .15 | .374 | .302 | .524 | .147 | .377 | .3 | .525 | .153 | .373 | .304 | .527 |
| PseudoR2 | .121 | .109 | .138 | .154 | .12 | .108 | .135 | .154 | .115 | .102 | .134 | .15 |
| Clusters | 675 | 685 | 685 | 685 | 671 | 685 | 685 | 684 | 677 | 685 | 685 | 685 |
| N | 106,331 | 106,847 | 106,970 | 106,892 | 104,071 | 104,629 | 104,757 | 104,677 | 159,585 | 159,856 | 159,947 | 159,869 |

Table A.18: Main result: including post

| | all observations | | | | semester of intervention | | | | semester after intervention | | | |
|---|---|---|---|---|---|---|---|---|---|---|---|---|
| | (1) beef | (2) poultry/fish | (3) veg | (4) meat | (5) beef | (6) poultry/fish | (7) veg | (8) meat | (9) beef | (10) poultry/fish | (11) veg | (12) meat |
| treated | -0.002 | -0.001 | 0.003 | -0.003 | -0.006 | -0.004 | 0.007 | -0.010 | -0.001 | 0.002 | 0.000 | 0.001 |
| | (0.005) | (0.008) | (0.007) | (0.008) | (0.005) | (0.008) | (0.008) | (0.008) | (0.006) | (0.010) | (0.010) | (0.011) |
| aftertreat=1 | 0.002 | -0.006 | 0.001 | -0.003 | 0.005 | 0.009 | -0.017 | 0.014 | 0.008 | -0.006 | -0.000 | 0.002 |
| | (0.006) | (0.008) | (0.008) | (0.008) | (0.010) | (0.013) | (0.012) | (0.013) | (0.008) | (0.011) | (0.010) | (0.012) |
| Mean of DV | .161 | .394 | .279 | .556 | .158 | .397 | .279 | .555 | .164 | .393 | .276 | .557 |
| PseudoR2 | .116 | .105 | .134 | .151 | .12 | .11 | .137 | .155 | .115 | .101 | .133 | .15 |
| Clusters | 681 | 685 | 686 | 685 | 676 | 685 | 686 | 685 | 677 | 684 | 685 | 685 |
| N | 199,716 | 199,756 | 199,963 | 199,835 | 126,780 | 127,209 | 127,394 | 127,292 | 156,727 | 156,962 | 157,087 | 157,008 |

# 2 Experiment Materials

## 2.1 Pamphlets

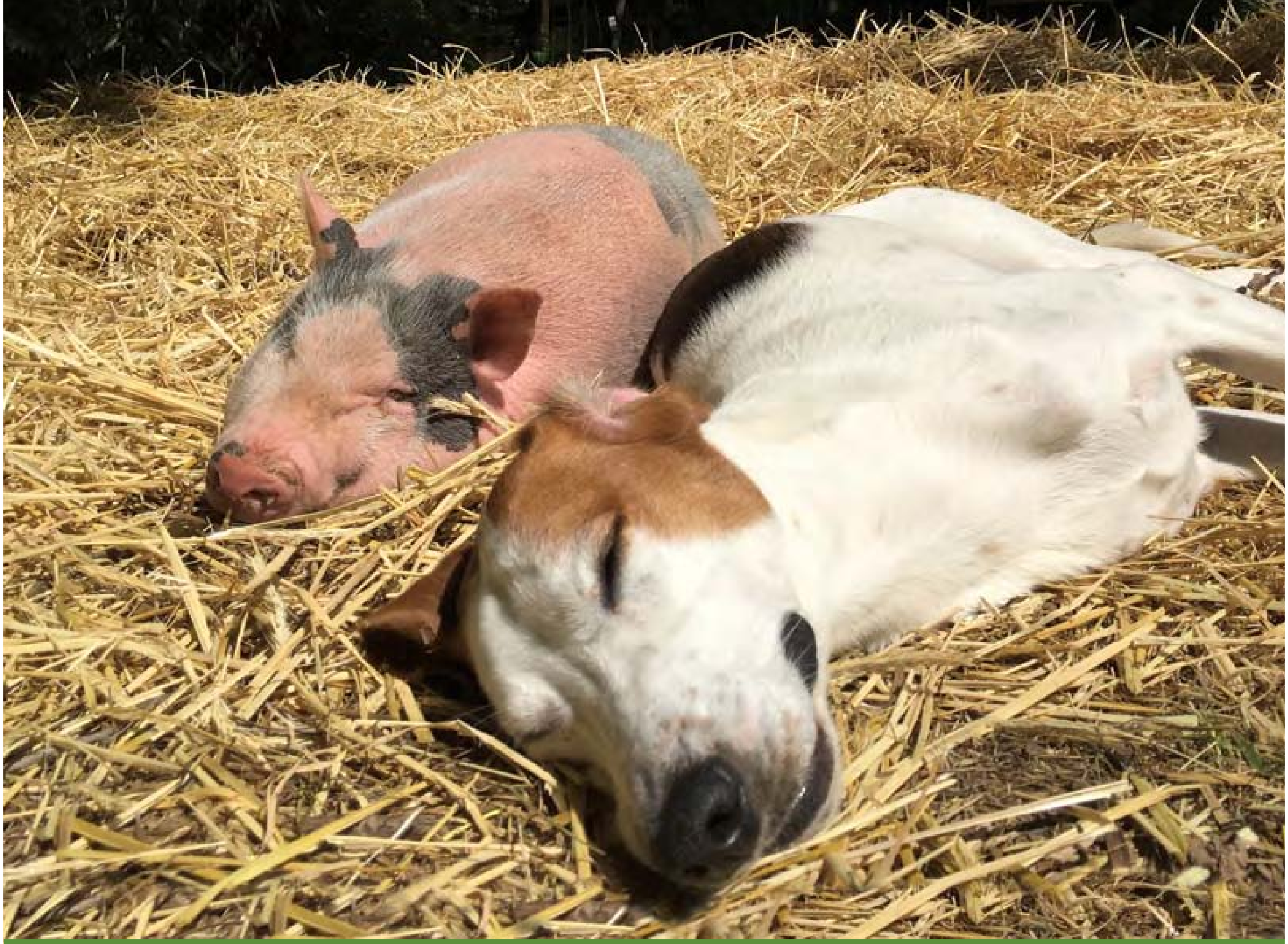

# COMPASSIONATE CHOICES

If You Care About Animals, Please Consider Not Eating Them

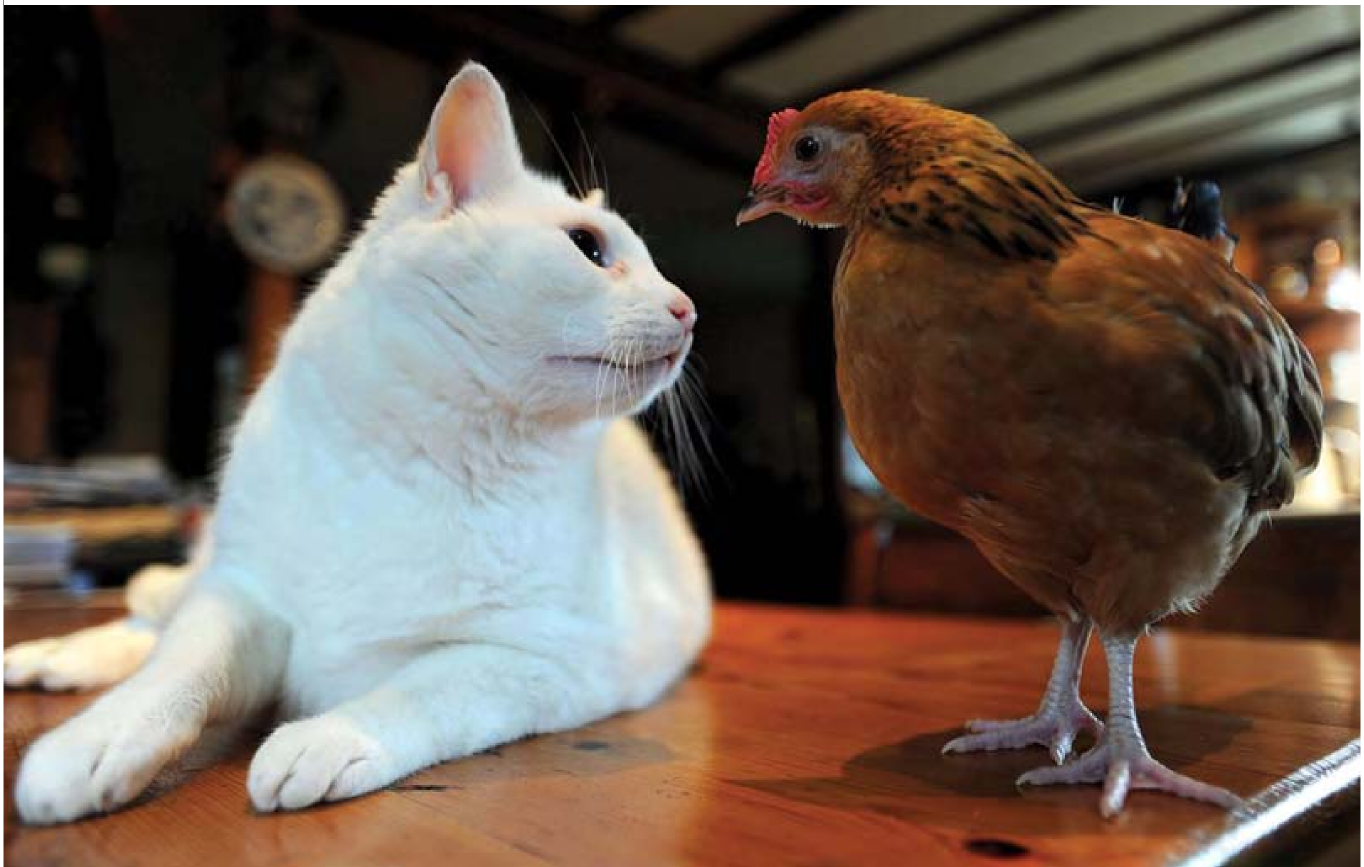

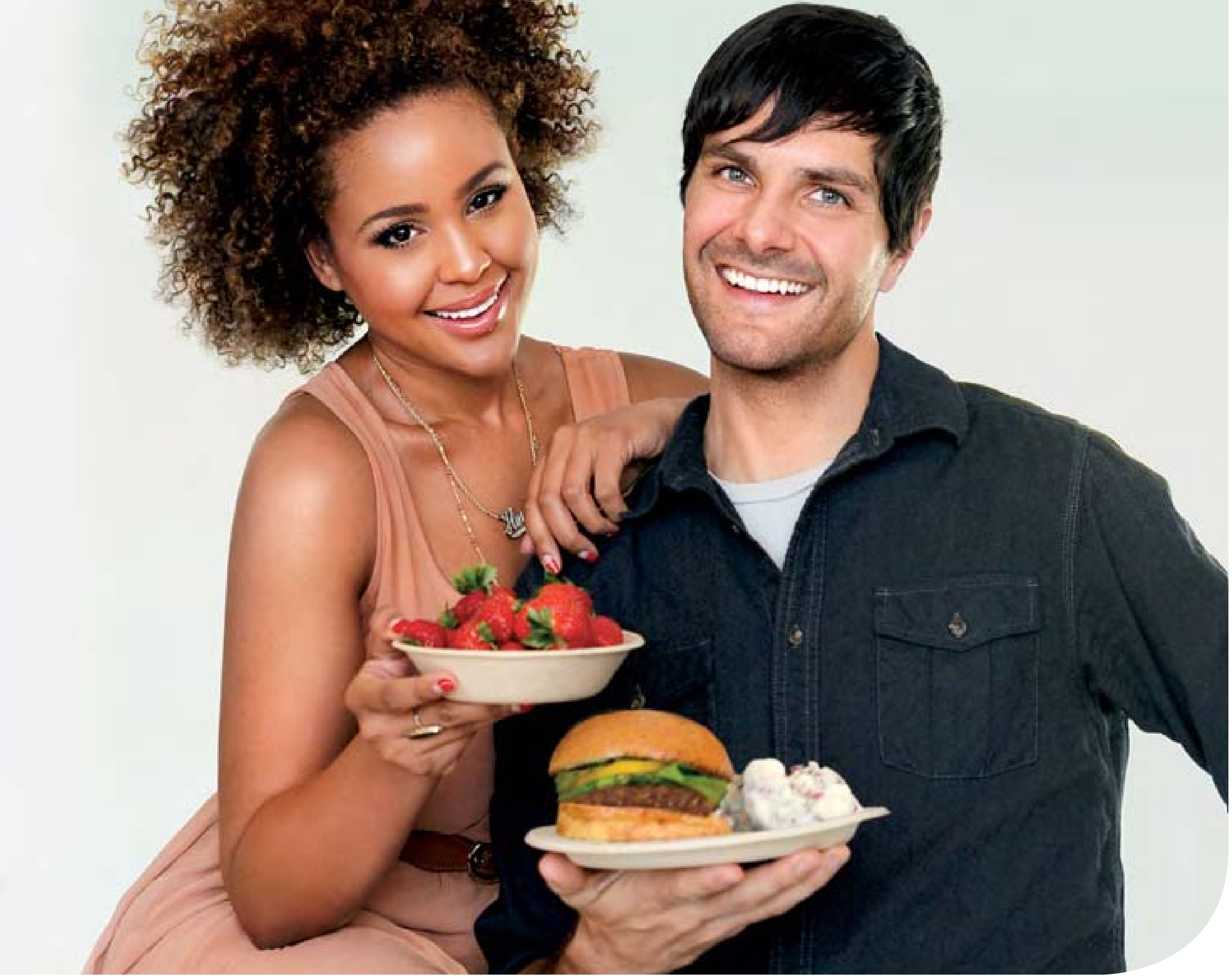

# A NATIONAL TREND

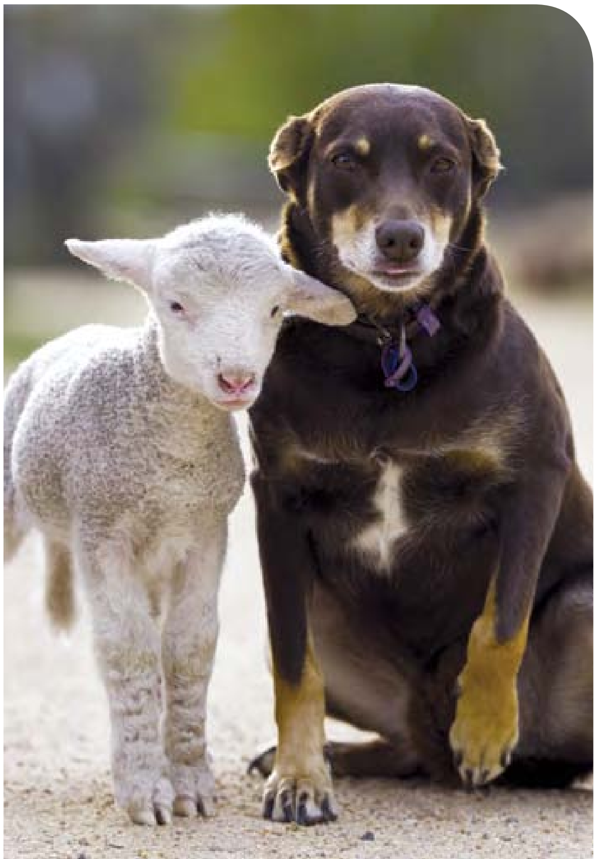

You may have already seen how animals are treated on today's farms. You even may have seen how they are killed in slaughterhouses. You probably don't like seeing those pictures and videos. Why? You're a kind and decent person. You don't want others to suffer unnecessarily.

More and more people like you are choosing to leave cows, pigs, chickens, fish, and other animal products off their plates.

Read on to see the ways that you can prevent animals from suffering by making changes to how you eat.

## A 2016 Harris Poll showed that about 3.7 million Americans are vegan—eating no meat, fish, dairy, or eggs. Millions more are vegetarian and don’t eat meat or fish.

Almost every animal-based food has animal-free alternatives that are delicious, satisfying, and available in most grocery stores—from high protein meat alternatives to cheese that melts on pizza to decadent desserts.

Whether you decide to cut back on meat—or remove all animal products—you can make a difference for animals at every meal!

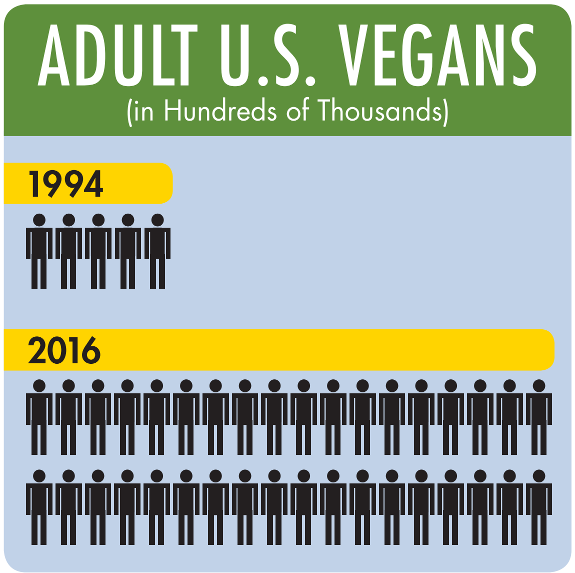


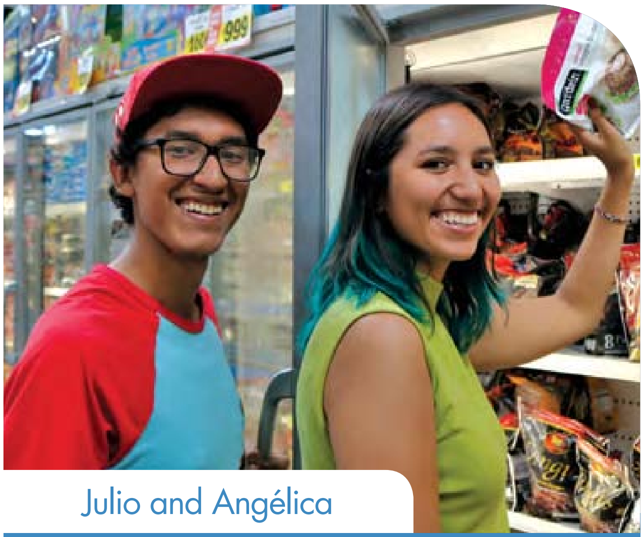

Julio and Angélica

“Thinking about how we love and care for our dogs and cats made us realize we shouldn’t be killing and eating farmed animals when there are now so many high protein, plant-based meats available in most grocery stores!”

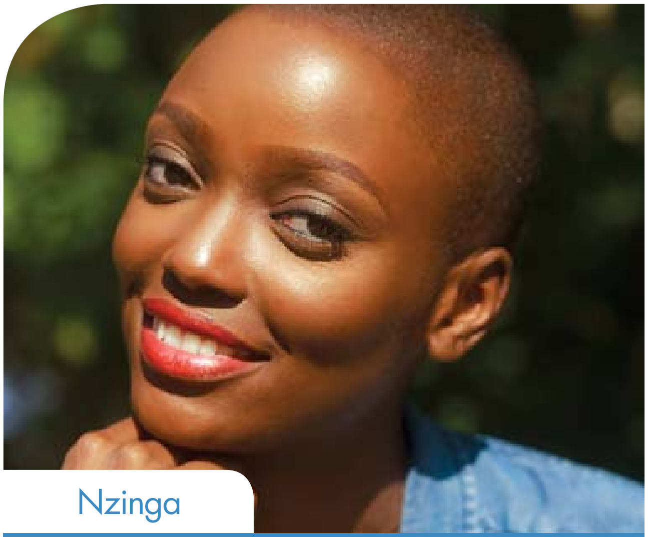

Nzinga

“I didn’t change overnight—I set a goal of going vegan and worked toward it every day. I chose the plant-based option whenever one presented itself and learned from leaflets like this. After a few months, I was completely vegan!”

> "Many of the nation's most routine animal farming practices would be illegal if perpetrated against cats and dogs."
>
> Jonathan Lovvorn, Chief Counsel, The Humane Society of the United States

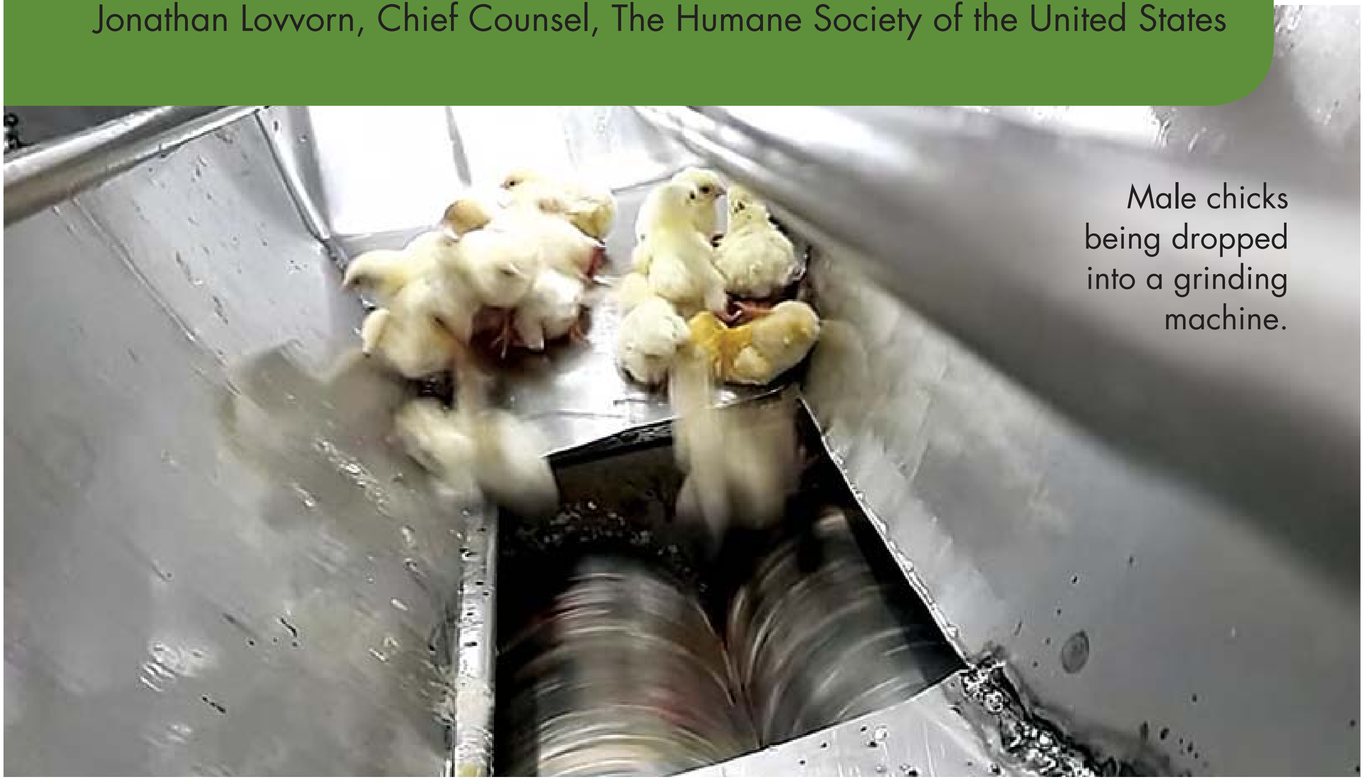

Male chicks being dropped into a grinding machine.

# MEET SCARLETT

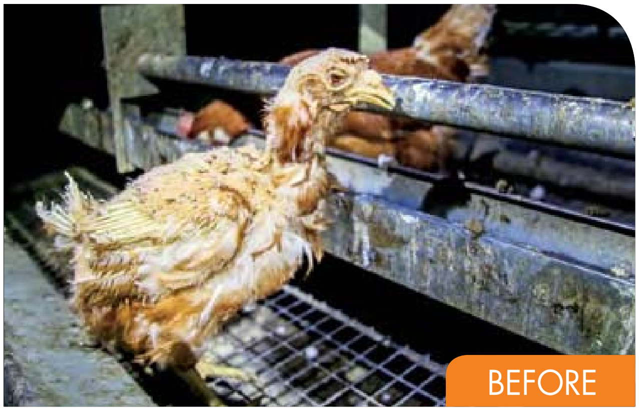


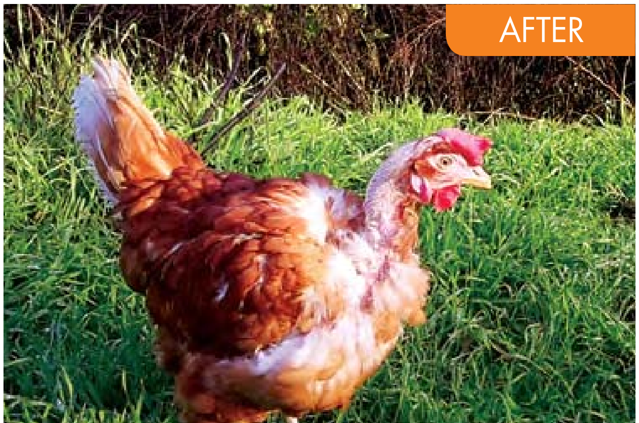


Like all chickens, Scarlett has a unique personality. Studies also show chickens have a sense of time and they anticipate the future.

Scarlett was raised for her eggs in a cage-free facility and was suffering terribly when she was rescued but now lives in a loving home.

Because egg farms—including free range and cage-free—have no use for male chicks, they are often tossed alive into a grinding machine (pictured above). Others are thrown into garbage bags to suffocate or starve.

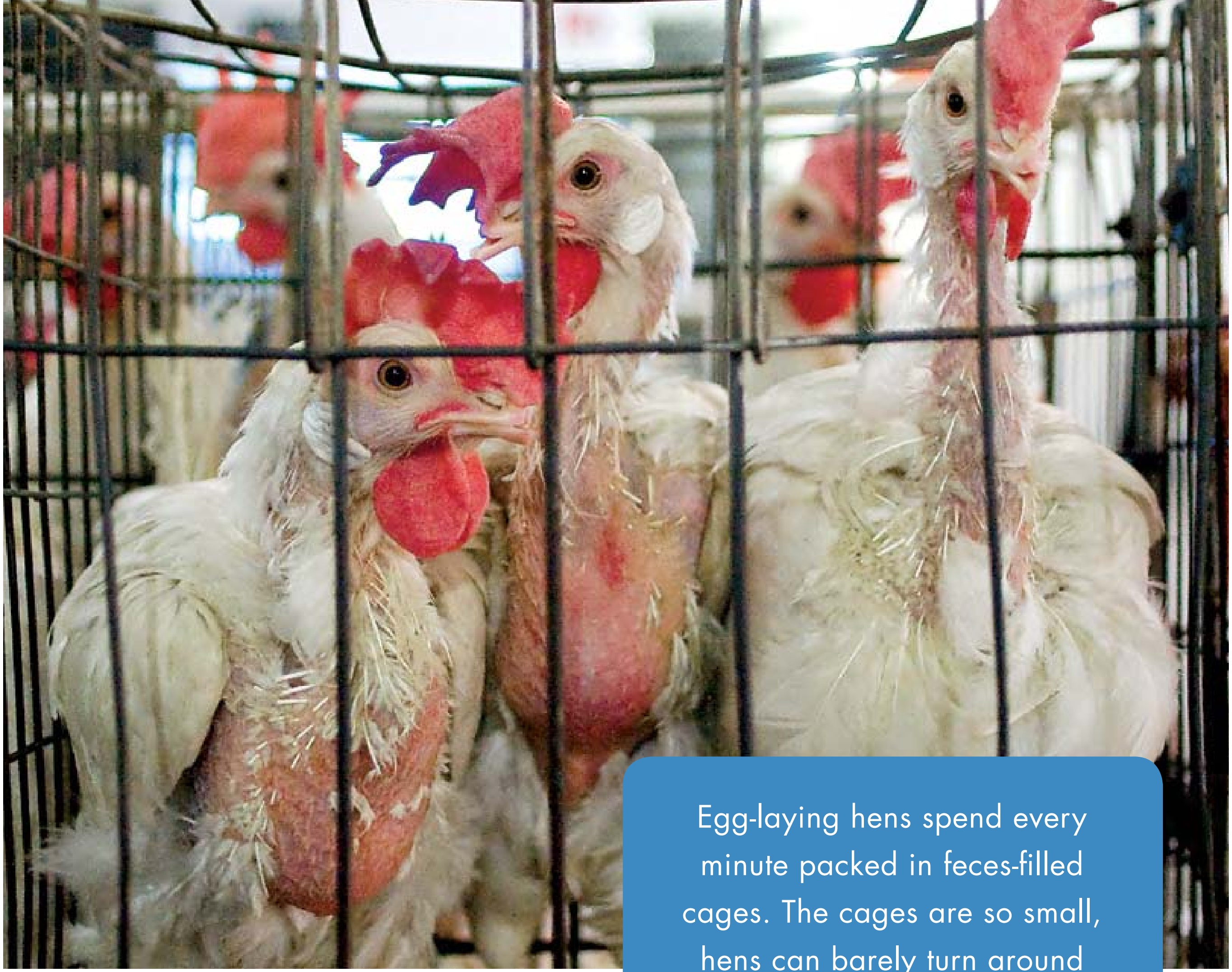

Egg-laying hens spend every minute packed in feces-filled cages. The cages are so small, hens can barely turn around and cannot spread their wings.

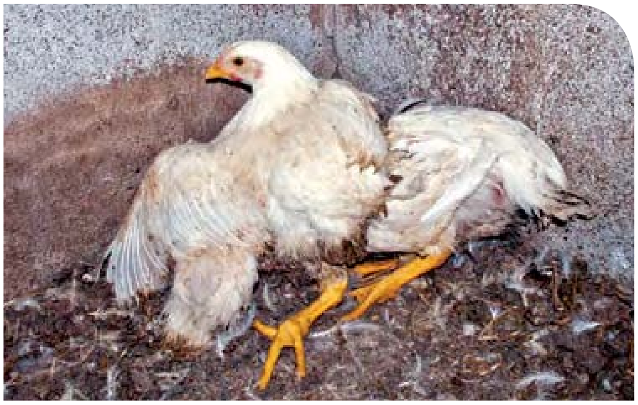

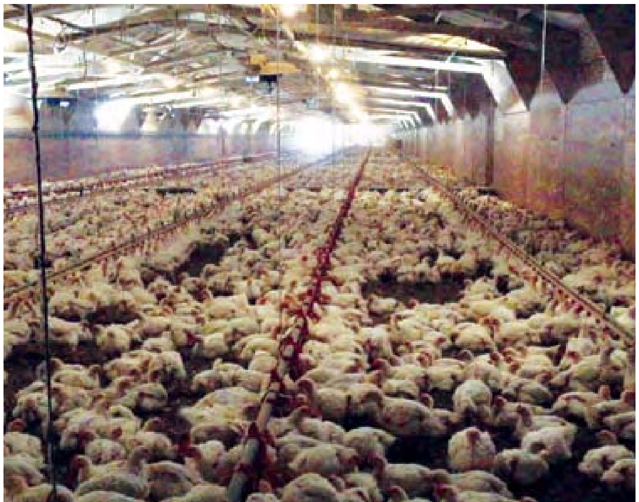

Chickens raised for meat spend their lives packed in a massive warehouse. They have been bred to grow so fast that by the time they are one month old it hurts many of them to walk. Ammonia from waste is so concentrated it burns their skin and lungs.

When chickens get sick, they can be clubbed on the head with a metal rod or left to suffer to death. At the slaughterhouse, they are electrically paralyzed before having their throats cut. If they avoid the blade—as many birds do—they will drown in a tank of scalding hot water.

"So our animals can't turn around for the 2.5 years they are in the stalls...who asked the sow if she wanted to turn around."

**Dave Warner,** Director of Communications, National Pork Producers Council

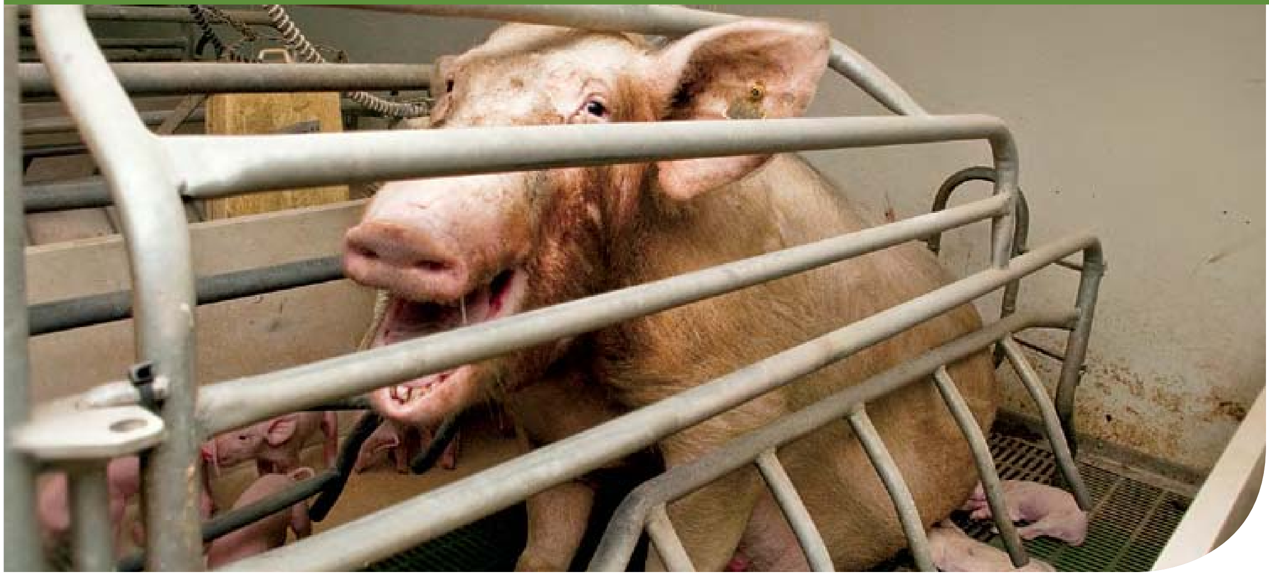

# MEET LUCILLE

Lucille (below) managed to flee from a transport truck on the way to auction. She ended up at Animal Place farmed animal sanctuary and is now living a peaceful life. Lucille loves belly rubs and comes when called—pigs are quite smart and perform as well as dogs and chimps in intelligence tests.

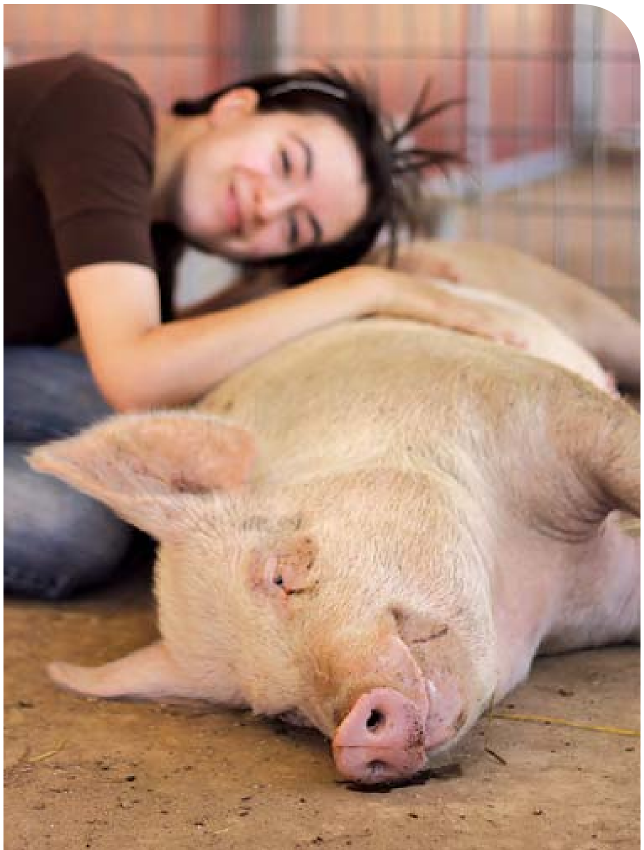

Mother pigs are typically kept confined in cages so small they cannot turn around (pictured above). In these cages, they develop severe psychological problems, such as biting the bars until their teeth break, and banging their heads.

Baby pigs are often born on metal grating. At just six months old, pigs raised for meat are electrocuted or shot in the head with a metal rod. Some don't immediately die and are drowned in scalding water.

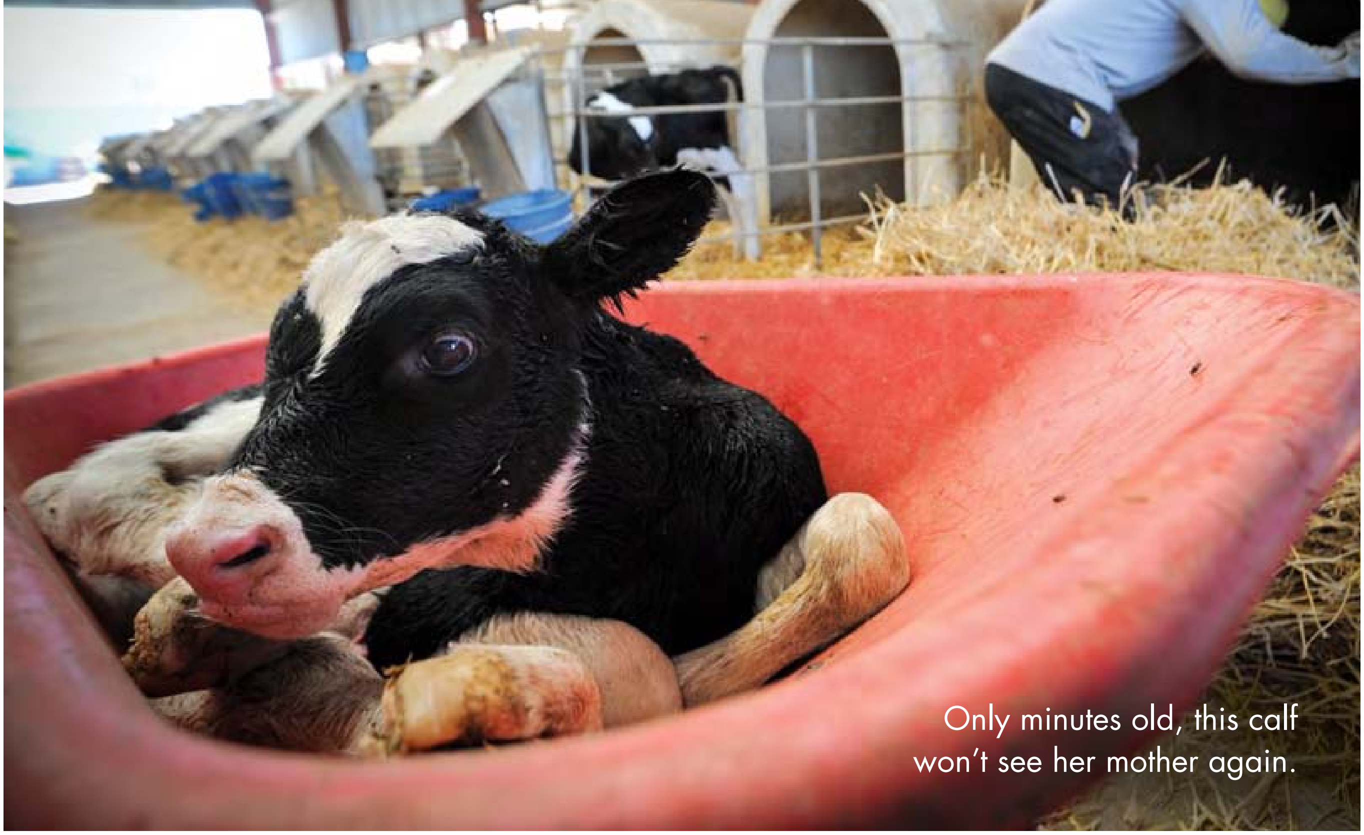
Only minutes old, this calf won't see her mother again.

# HOW DAIRY HARMS COWS

From 1940 to 2015, average milk production from a U.S. dairy cow rose from 2 to 11 tons per year. Producing so much milk leads to udder enlargement and breakdown. Cows can suffer from foot problems due to the conditions (pictured right).

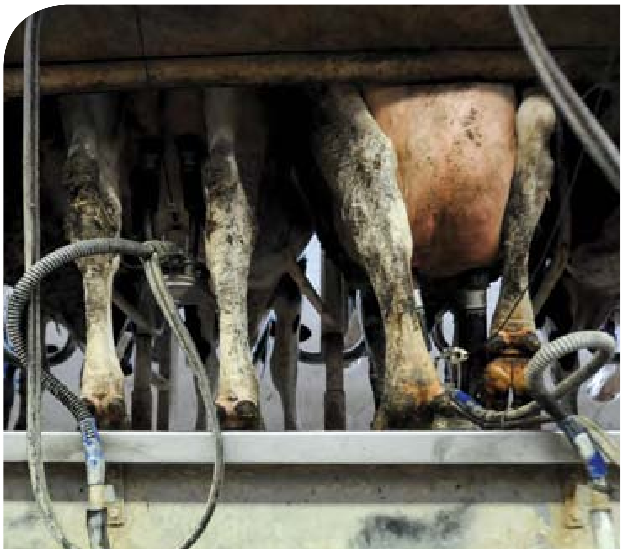

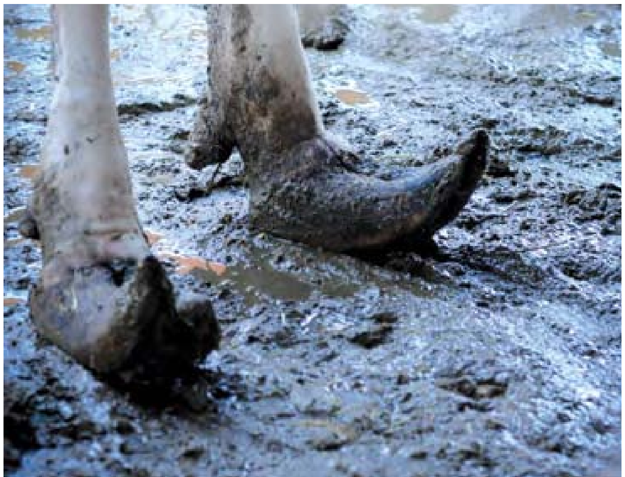

In order to produce profitable amounts of milk, a cow must be impregnated on a yearly basis. While the bond between mother and baby is one of the strongest in nature, dairy calves are taken away within hours of birth—they won't be together again.

The normal lifespan of a cow is twenty years, but modern dairy cows are slaughtered at about five, when their milk production starts to decline.

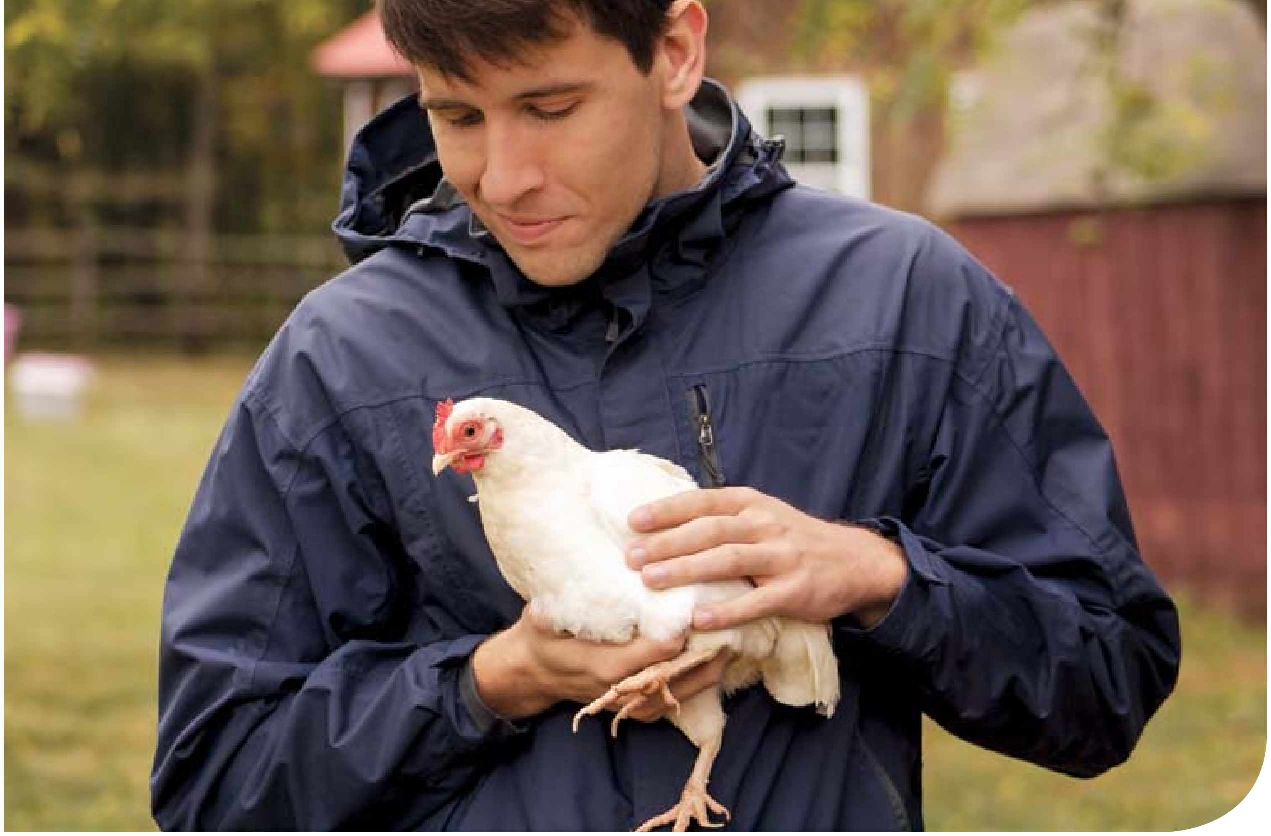

# MEET THE ONES YOU SPARE

Agricultural economists have found that when people eat less meat, producers raise and kill fewer animals. Here are some of the individuals you help spare each year.

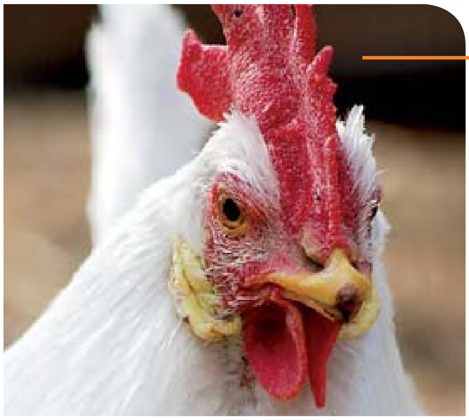

21 chickens like Tilly, and even more if you also cut out eggs

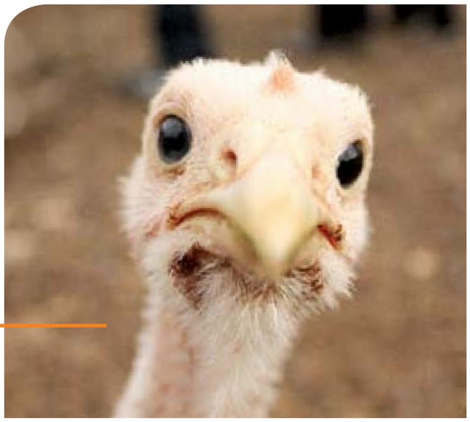

A turkey like Clove

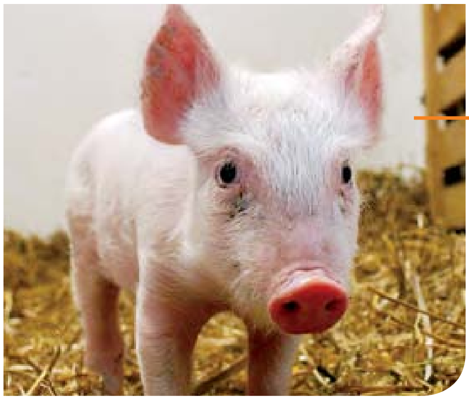

A pig like Bob Harper

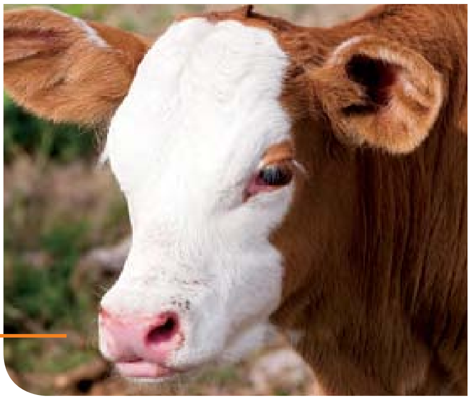

A cow like Meghan

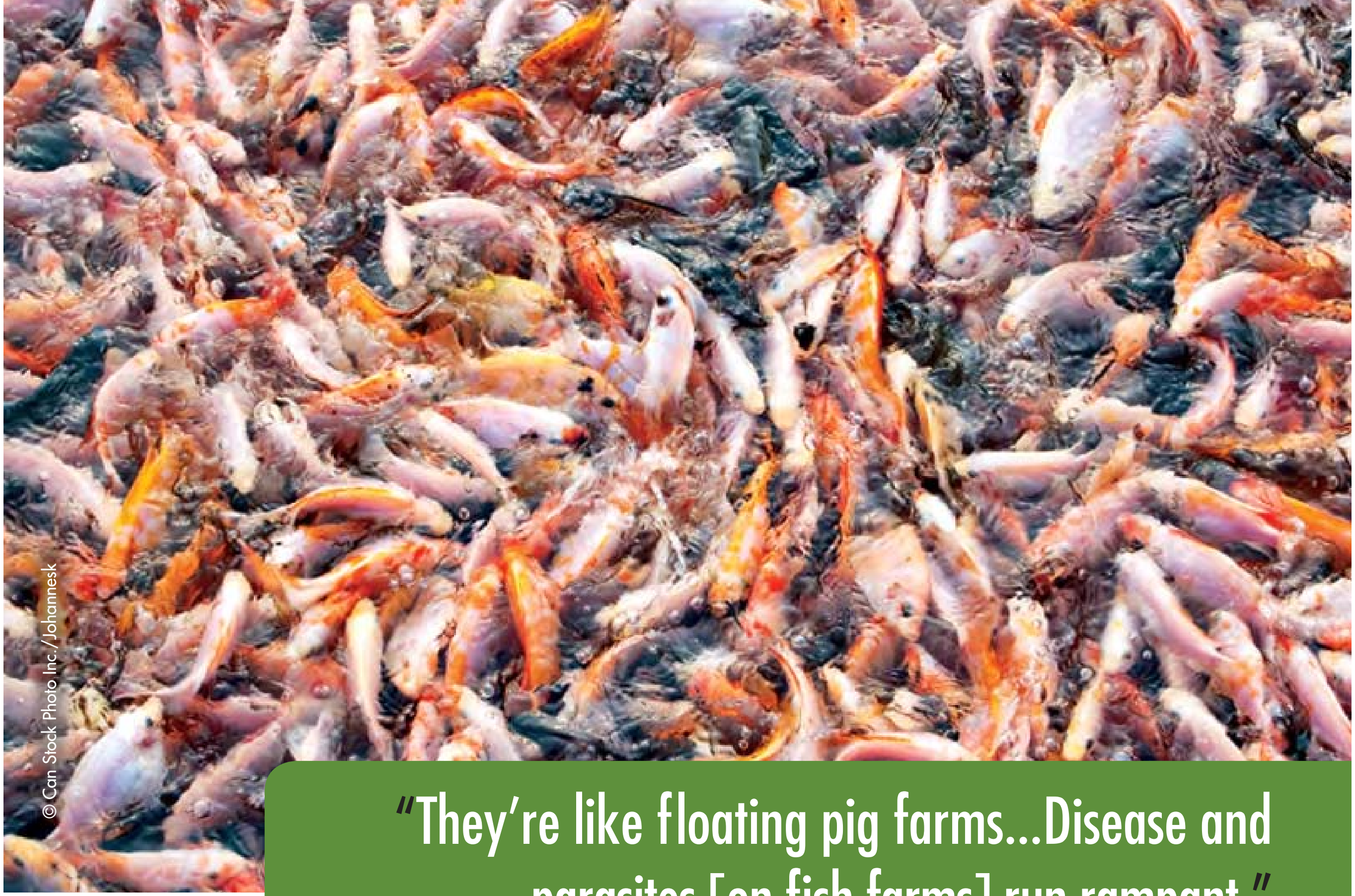

© Can Stock Photo Inc./Johannesk

"They're like floating pig farms...Disease and parasites [on fish farms] run rampant."

*The Los Angeles Times*, "Fish Farms Become Feedlots of the Sea"

About half of the fish consumed by humans don't come from the wild. Fish farms are often crowded enclosures where stress and filthy water cause death and disease. The manure drifts into neighboring rivers and oceans creating areas that no longer sustain life.

In the ocean, large driftnets catch everything in their path, including sea turtles, sharks, whales, and dolphins whose bodies are then discarded.

Fish pulled from the water suffocate for up to ten minutes. When dragged from deep ocean waters, their eyes bulge and their stomachs turn inside out from the change in pressure.

Fish have long-term memories and are smart enough to learn tricks. Like all farmed animals, fish are badly abused—they just can't cry out.

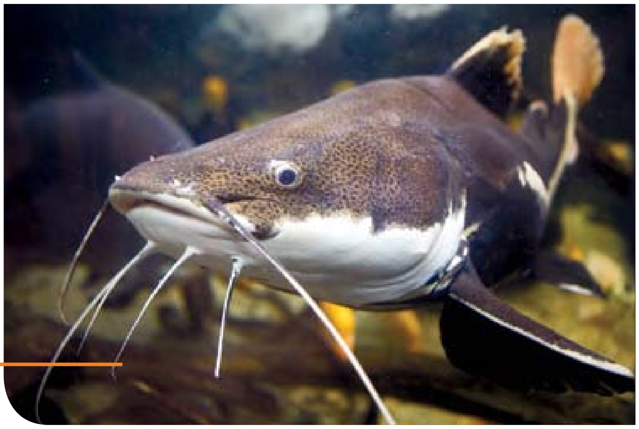

Dozens of fish
like this beautiful catfish

# HEALTH BENEFITS

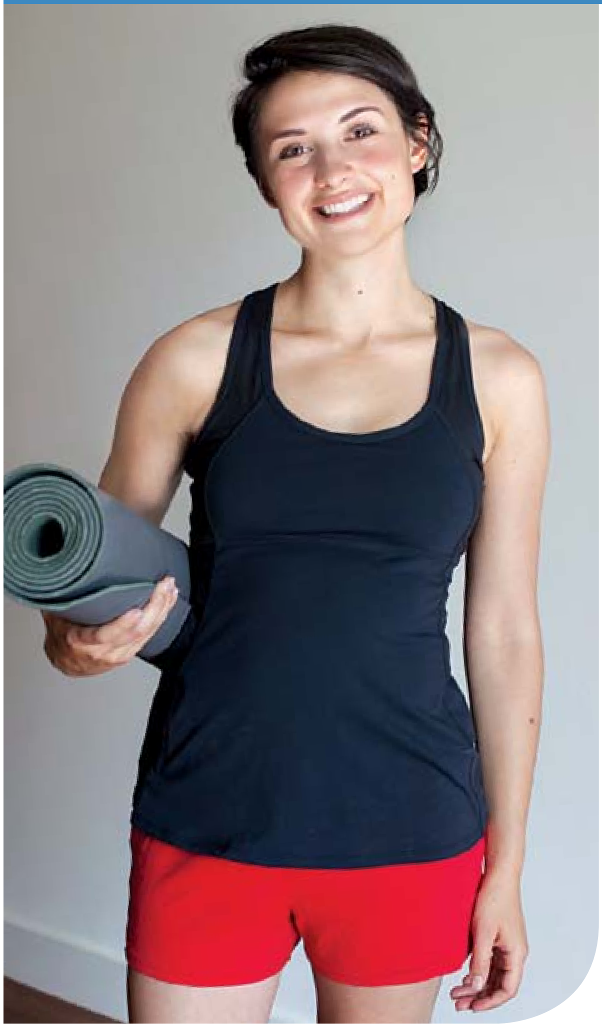

The Academy of Nutrition and Dietetics, the largest organization of nutrition professionals in the world, says that eating vegetarian or vegan has many benefits and is safe for people of all ages, including pregnant women.

While type 2 diabetes has become a health crisis, vegans are much less likely to develop this disease. Eating fewer animal products often results in lower cholesterol, lower blood pressure, and a reduced risk of cancer.

Many elite athletes and bodybuilders are vegan. You can order our *Compassionate Athlete* booklet at VeganOutreach.org.

"I can honestly say that being vegan is not only the most efficient way to be full-body strong, it's also the most humane."

**David Carter,** former NFL Player

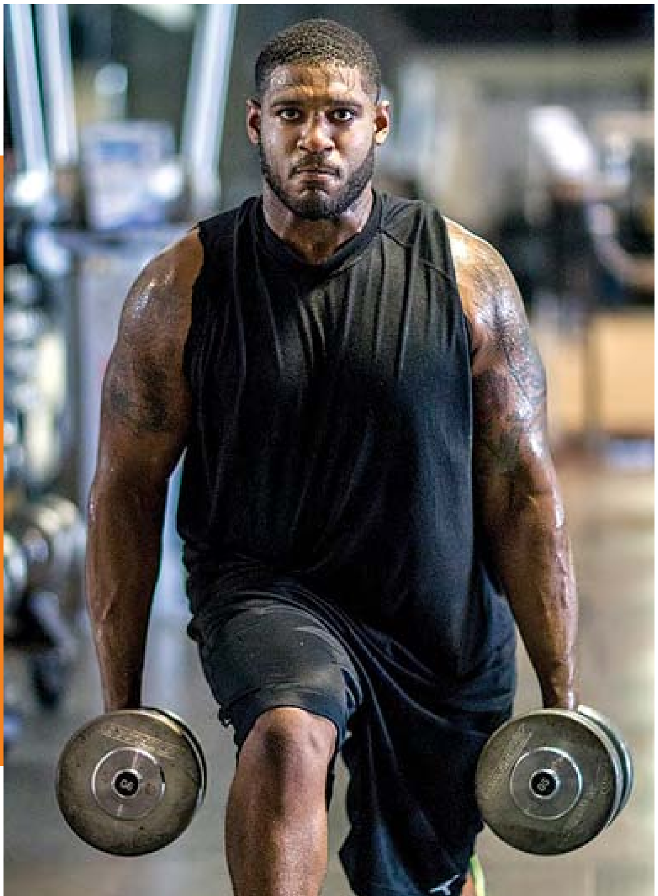

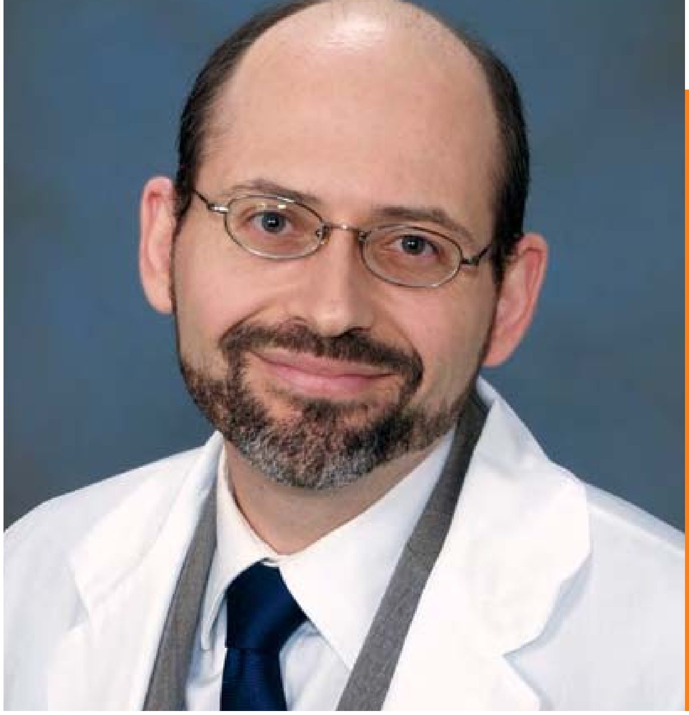

"As a medical doctor, I consider adopting a plant-based diet to be one of the most important things someone can do to prevent the leading causes of disease."

**Dr. Michael Greger,** NutritionFacts.org

# DOING IT RIGHT

Eating high protein foods such as beans, peanuts, and vegetarian meats will fulfill your daily protein requirement and provide satisfying meals. Plant-based diets are high in iron, and eating vitamin C at meals helps you absorb it. Consider a multivitamin with B12 to cover your bases. Order our *Guide to Animal-Free Eating* (see back cover) or visit VeganHealth.org for nutrition tips.

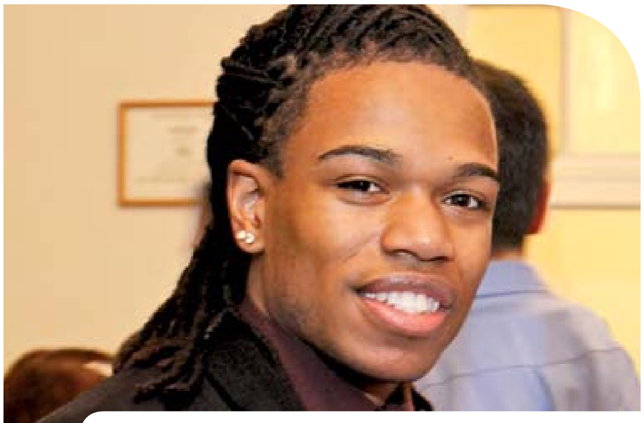

Brandon Williams, Temple University

"I lift five days a week, and I've added muscle since going vegetarian. Getting enough protein is easy. Beans, lentils, nuts, plant-based meats, tofu, whole grains, and dairy-free protein shakes and bars are all great sources of protein."

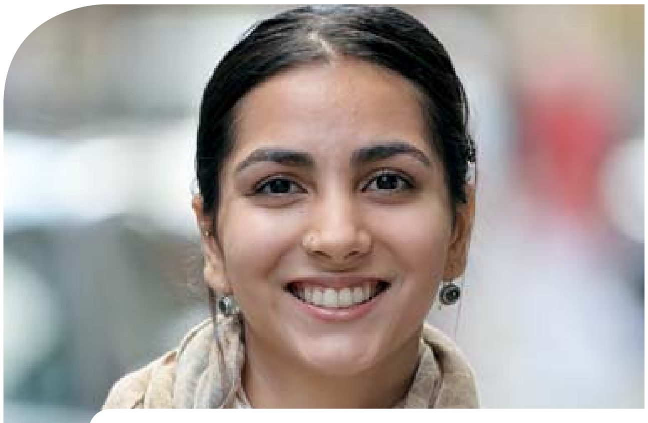

Gunita Singh, Boston University

"I've always been low on iron, even when I ate meat. So I make sure to eat foods with plenty of iron. Beans, dark leafy greens, and whole grains like oatmeal and wheat are filled with iron. There are also iron supplements out there if you need them."

# EATING OUT

## INTERNATIONAL RESTAURANTS

### ITALIAN

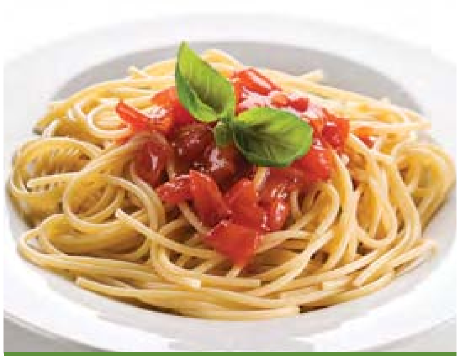

### THAI

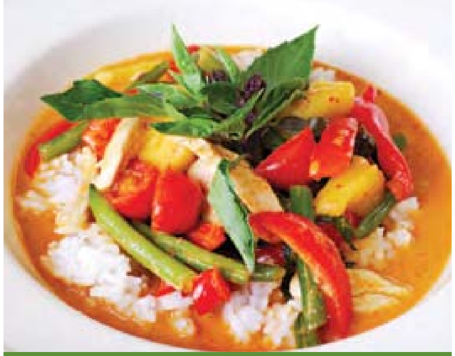

### MIDDLE EASTERN

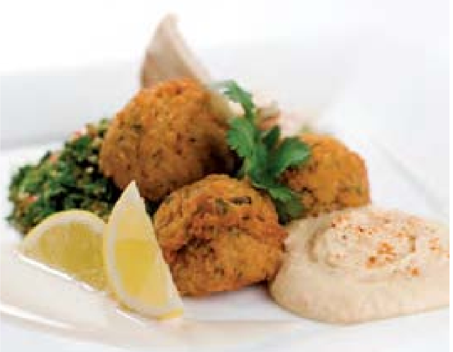

### INDIAN

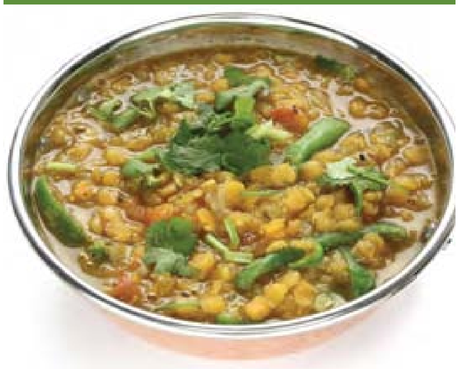

### MEXICAN

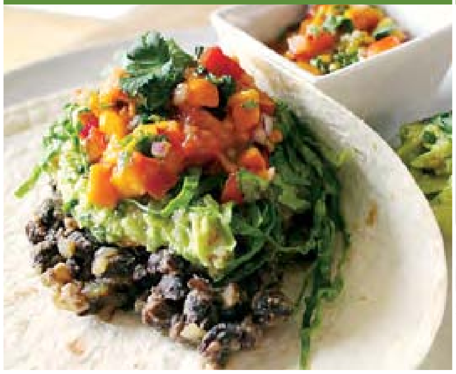

### CHINESE

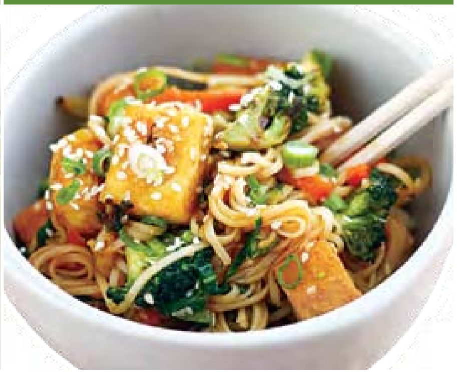

## CHAIN RESTAURANTS

- TACO BELL – bean burritos, bean tacos, potatoes, guacamole
- CHIPOTLE – burrito, bowl, or tacos with sofritas and fajita veggies
- JOHNNY ROCKETS – Streamliner burger and fries
- OLIVE GARDEN – pasta with marinara, breadsticks, minestrone soup
- NOODLES & COMPANY – Japanese pan noodles, spaghetti with marinara

## VEGAN PIZZA GALORE!

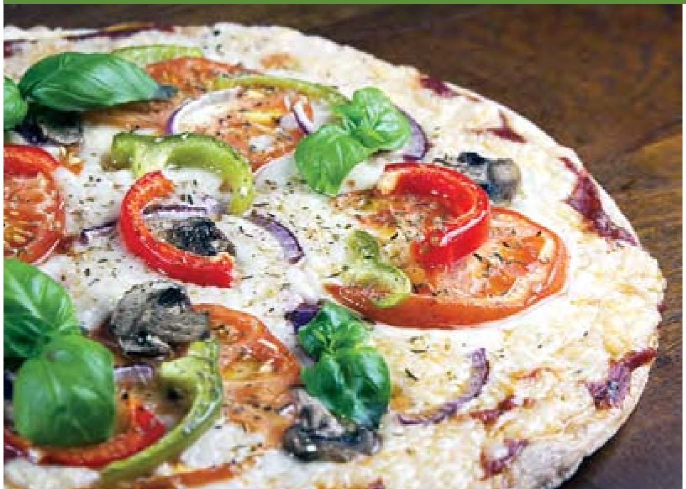

While cheeseless pizza with lots of fixings is always a tasty option, many chains now carry vegan cheese and meats!

Check out Mellow Mushroom, Pie Five, PizzaRev, MOD Pizza, Pieology, Pi Pizzeria, and many more!

# EASY MEAL IDEAS

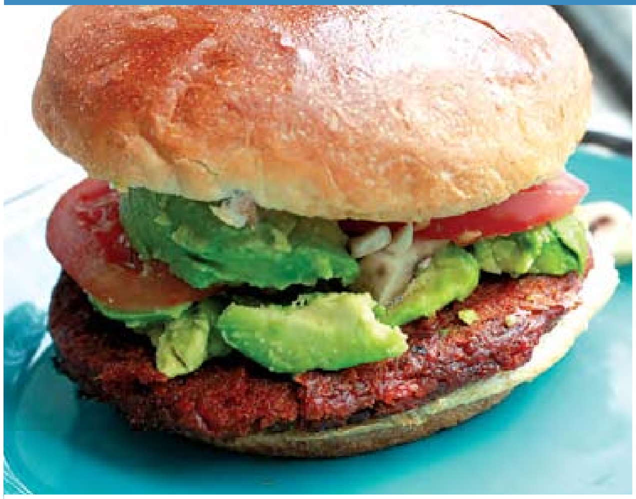

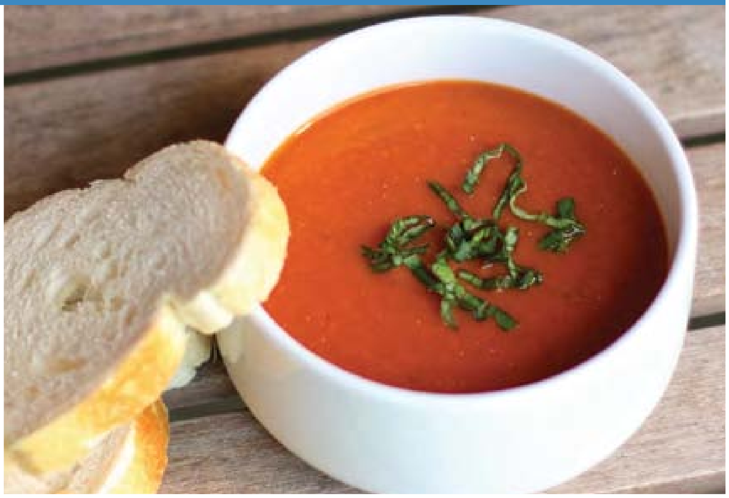

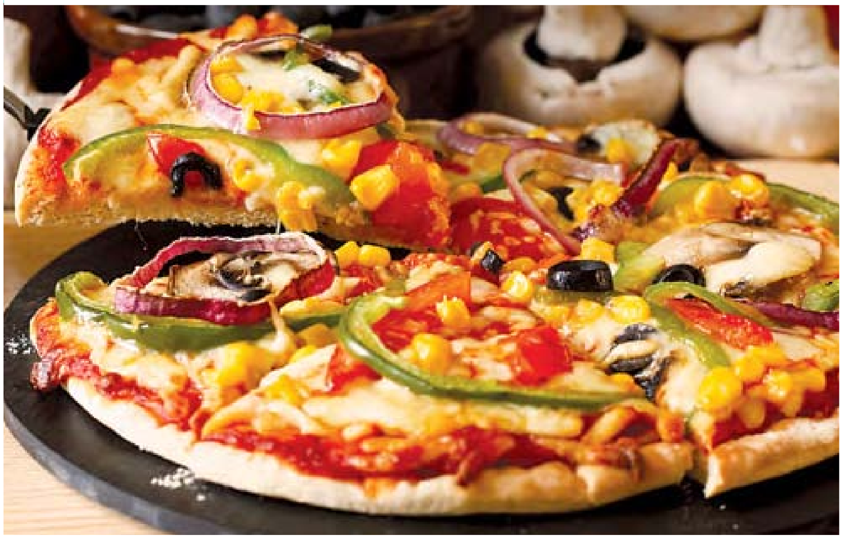

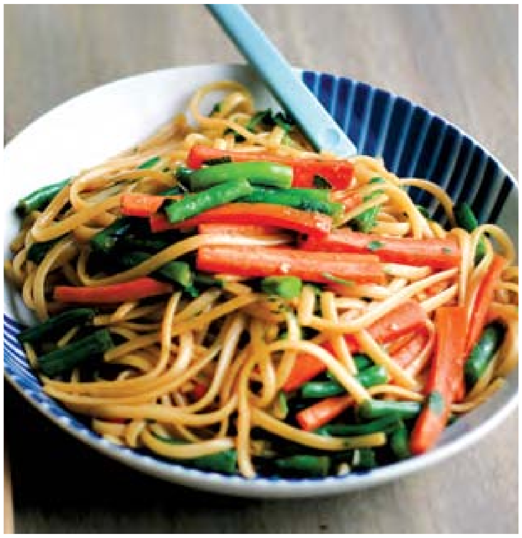

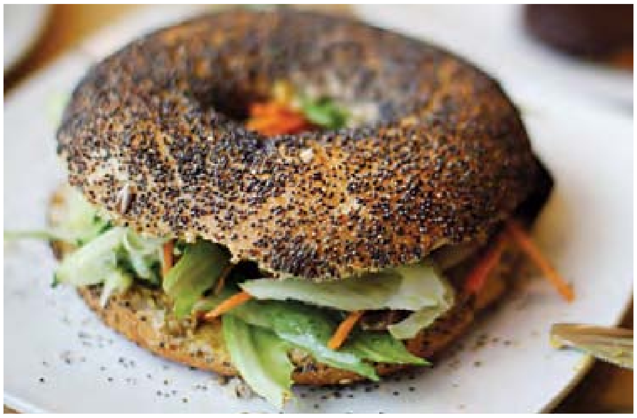

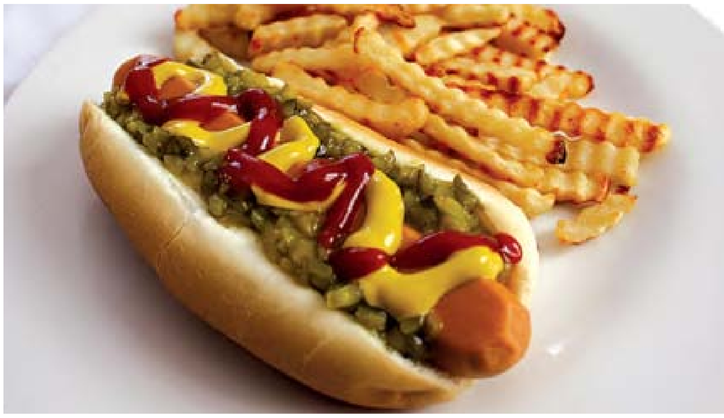

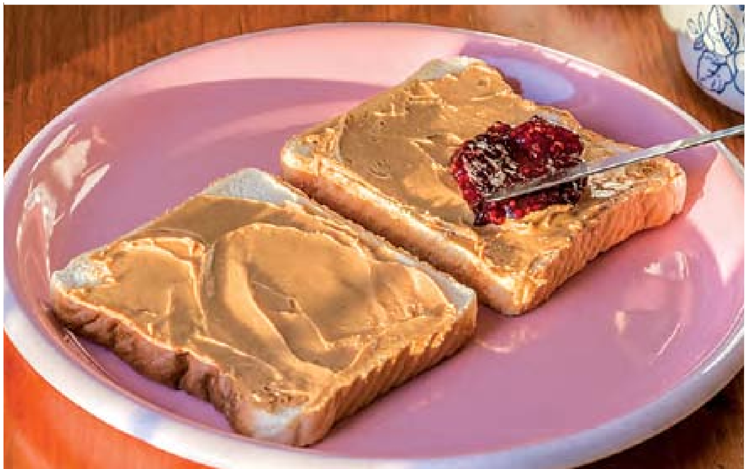

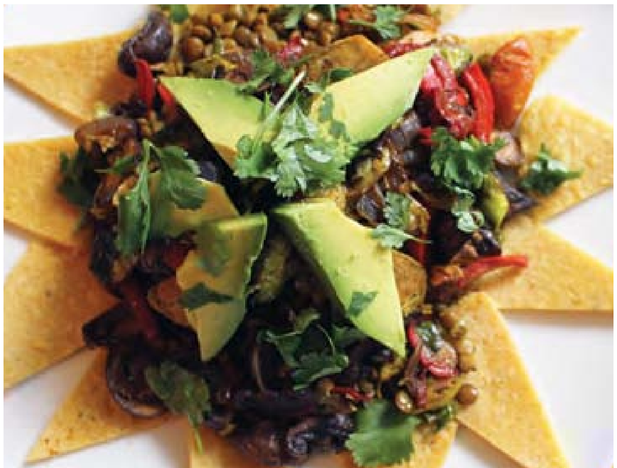

# GETTING GROCERIES

## PANTRY

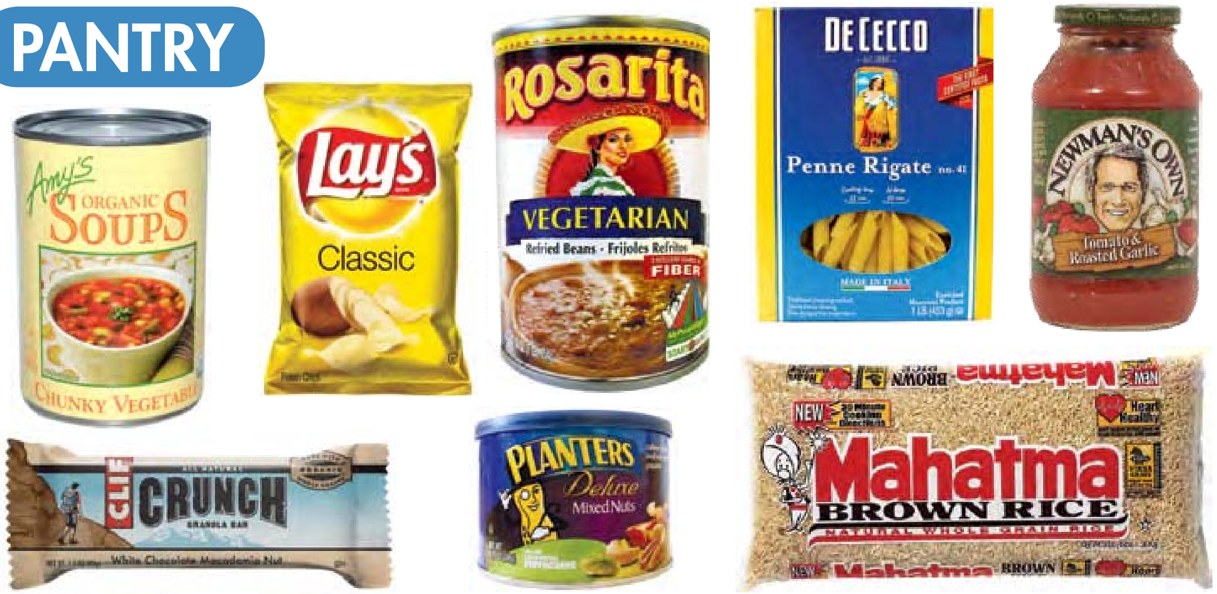


## FRIDGE

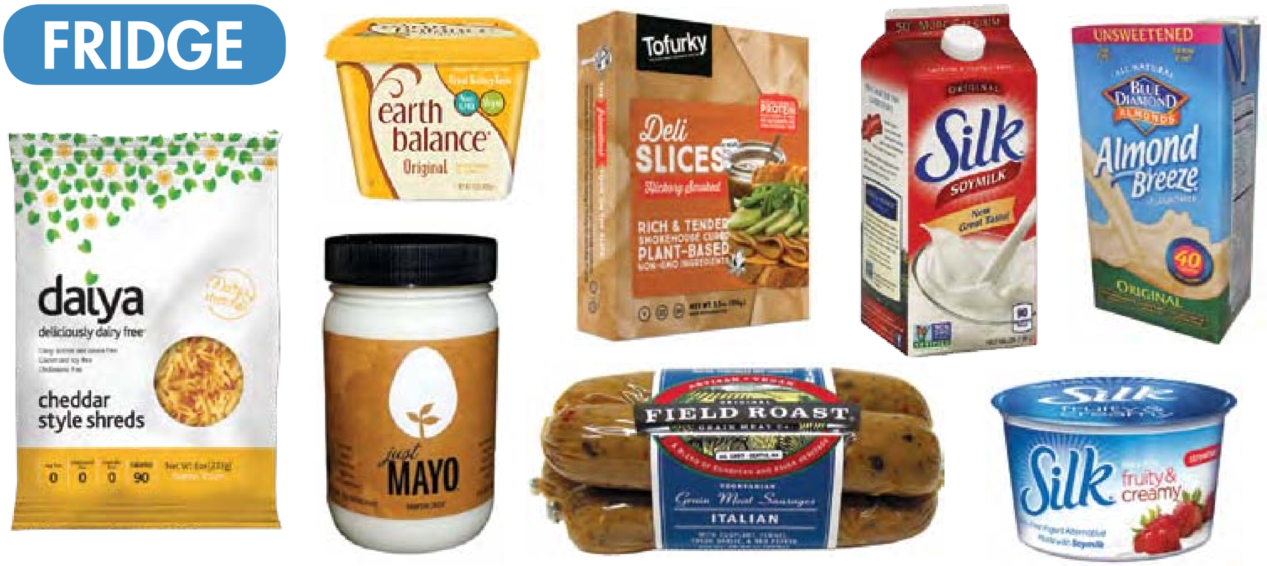


## FREEZER

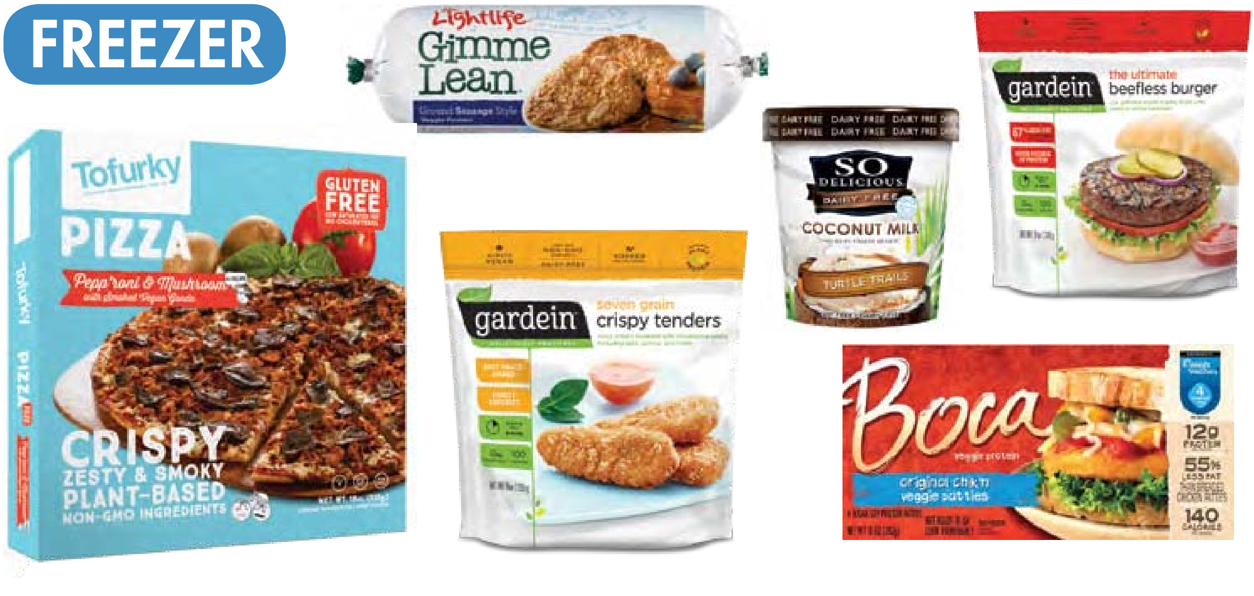

# MAKE A DIFFERENCE!

You can replace animal products with something better!

Research shows that people who make a more gradual transition to eliminating animal products are more likely to stick with it.

Because many more chickens are killed to produce the same amount of meat as from cows and pigs, you'll prevent more animal suffering by first eliminating chickens.

You don't have to sacrifice your favorite meals—high protein vegan meats are widely available.

Focus on the hundreds of new foods you can add to meals—include them in your routine until there's no room left for the old animal products!

Thank you for caring about the suffering of individuals who do not have the power to stand up for themselves!

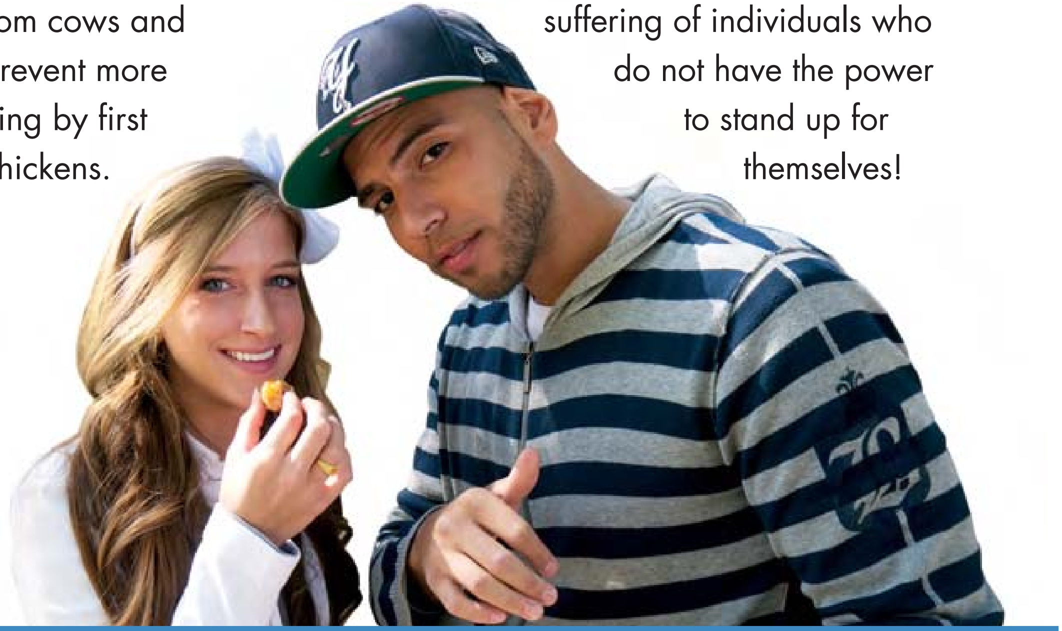

**Once you've decided what will work for you, just get started and stick with it!**

VEGAN OUTREACH

PO Box 1916, Davis, CA 95617 • VeganOutreach.org/Contact

Photo copyrights: © Diana Dauer (cover upper); Archant CM Ltd (cover lower); Melissa Schwartz (p2 upper); Edgar's Mission (p2 lower); Katia Rodríguez (p3 lower left); M. Cameron Crew (p3 lower right); Anonymous for Animal Rights, Israel (p4 upper); Direct Action Everywhere (p4 lower); Humane Society of the United States (p5 upper); Mercy For Animals (p5 middle, bottom); Animal Equality (p6 upper); Adrienne Szamotula (p6 lower); Jo-Anne McArthur (p7 all); Tatiana Mendez (p8 upper); Farm Sanctuary (p8 chicken, pig); Toni Okamoto (p8 turkey); CC SA BYNC Casey Morris (p8 cow); Can Stock Photo Inc/Johannesk (p9 upper); Art Phaneuf Photography (p9 lower); Stephanie Crumley (p10 upper); Paige Lynn Photography (p10 lower); iStock.com/dulezidar (p12 Italian); iStock.com/SochAnam (p12 Thai); iStock.com/kcline (p12 Middle Eastern); Shutterstock/bonchan (p12 Indian); Brownble.com (p12 Mexican); ilovevegan.com (p12 Chinese); Paige Lynn Photography (p10 lower); iStock.com/dulezidar (p12 Italian); iStock.com/SochAnam (p12 Thai); Stock.com/kcline (p12 Middle Eastern); Shutterstock/bonchan (p12 Indian); Brownble.com (p12 Mexican); Stephanie Crumley (p10 upper); Follow Your Heart (p12 lower); (CC) SweetOnVeg (p13 veggie burger); Margaret Chapman (p13 soup); iStock.com/StockSolutions (p13 pizza); Stephanie Lundstrom (p13 stir-fry); (CC) Suzutte (p13 bagel); (CC) Alex in Leeds (p13 veggie dog); (CC) Mateeas/Matias Garabedian (p13 PBJ); Renee Press (p13 nachos); Farm Sanctuary (back cover upper)

© 2017 Vegan Outreach

VeganPrinter

printreleaf CERTIFIED Printed with vegetable inks.

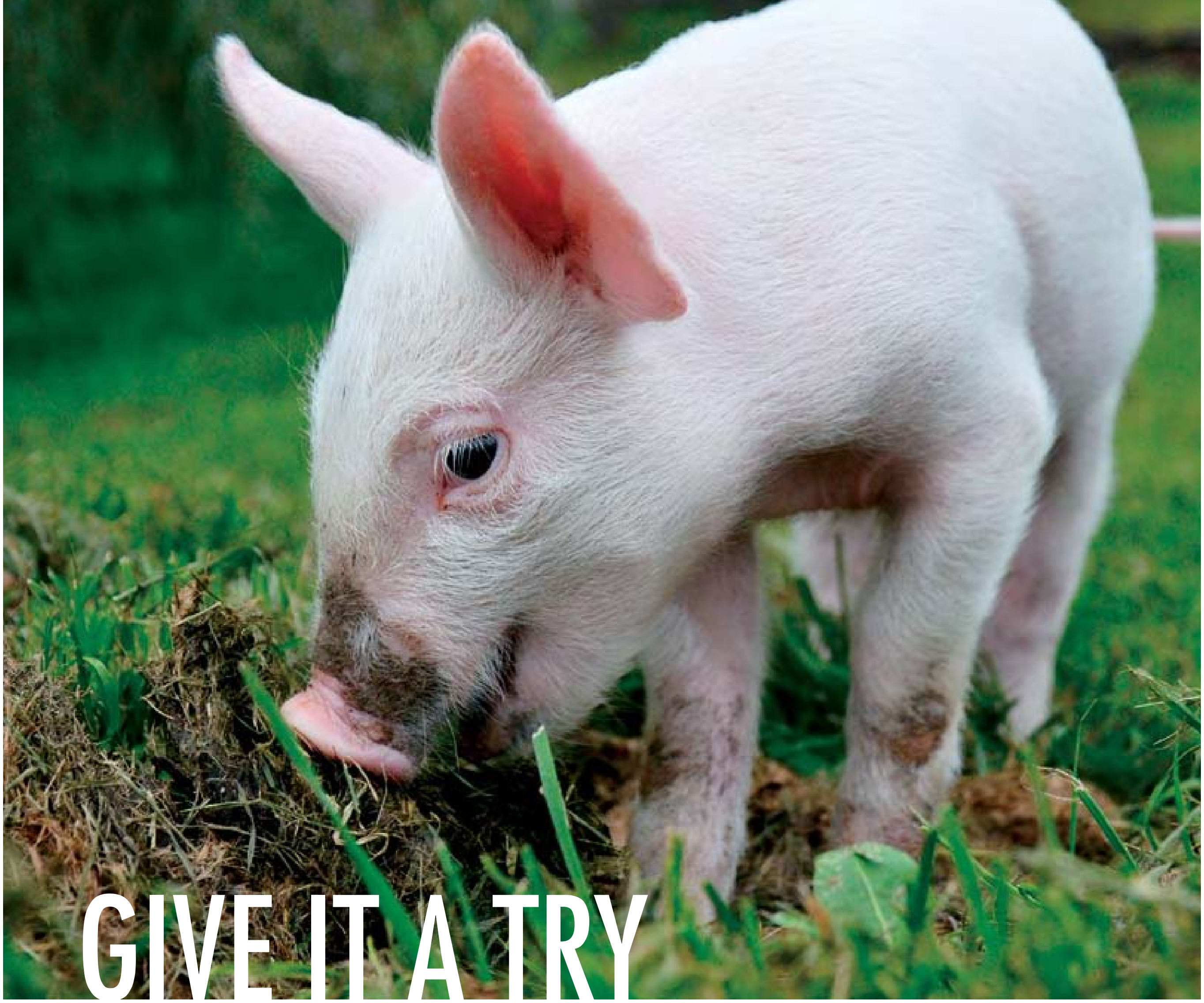

# GIVE IT A TRY

## FREE GUIDE

Get your animal-free eating guide with recipes and health tips:

Text: "Starter" to 55678

Visit: VeganOutreach.org/Guide

## MENTOR PROGRAM

Get free individual help in going veg:

VeganOutreach.org/VMP

## MORE COPIES

To spread this information:

VeganOutreach.org/Order

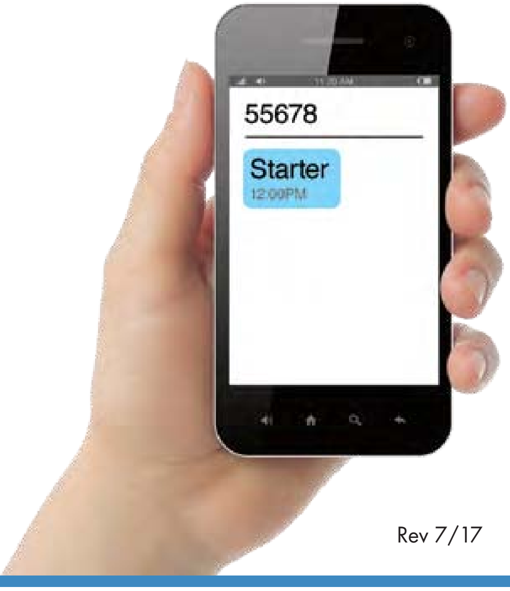


What do you think of this booklet? Let us know:

VeganOutreach.org/Contact

Rev 7/17

Follow us on Facebook, Instagram & Twitter

# The CRUELTY Behind the Cuteness

## You Can Stop It

The dog breeder or pet store you've picked to buy your puppy from might be supporting the notorious "puppy mill" industry. Puppy mills are breeding businesses that raise dogs in shockingly poor conditions. "Breeding stock" animals are caged and continually bred for years, without human companionship and with little hope of ever becoming part of a family. After their fertility wanes, breeding animals may be killed, abandoned, or sold to another mill. The result of all this breeding? Millions of puppies, many with behavior and health problems not easily seen at the time of purchase.

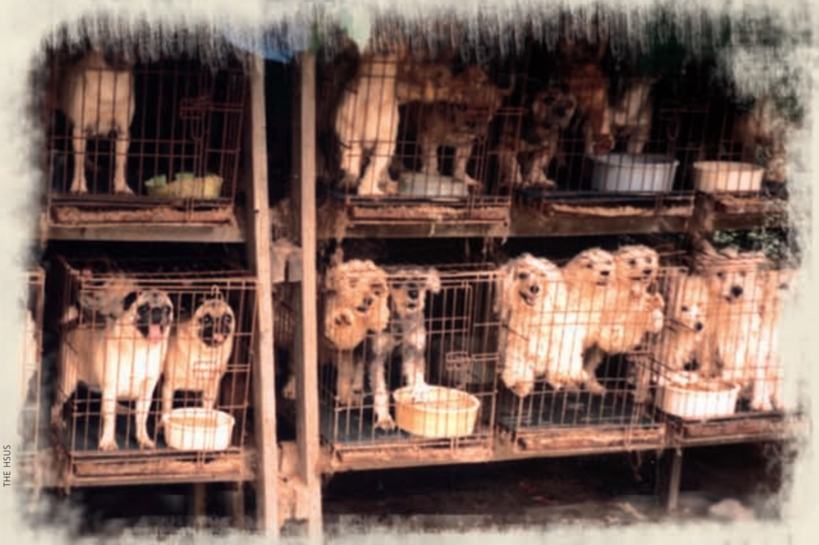

THE HSUS

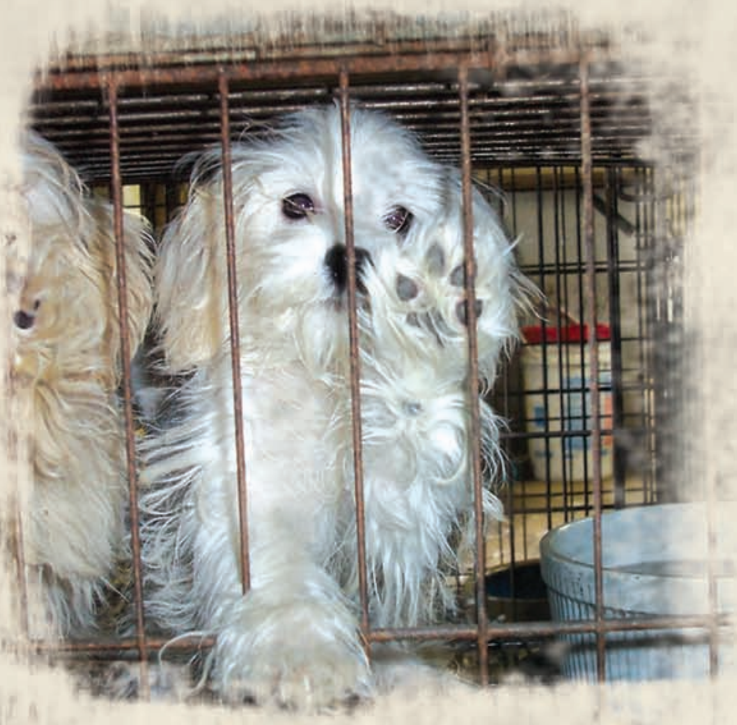

## Without Pet Stores, Puppy Mills Wouldn't Survive

They may seem to know what they're doing, but behind the friendly façade of pet stores often lies the ugly reality of puppy mills. There's only one sure way to combat the tragedy of puppy mills—don't support them. No matter how cute the puppy in the pet store is, please don't buy her. You may feel like you're "rescuing" her, but in reality you're only freeing up space for another puppy mill "product" while supporting and encouraging an industry based on abuse. Unless you personally visit the place your puppy was born and raised—and where the puppy's parents live—there's no way to know that your puppy didn't come from a puppy mill, no matter what a sales clerk tells you. ➜

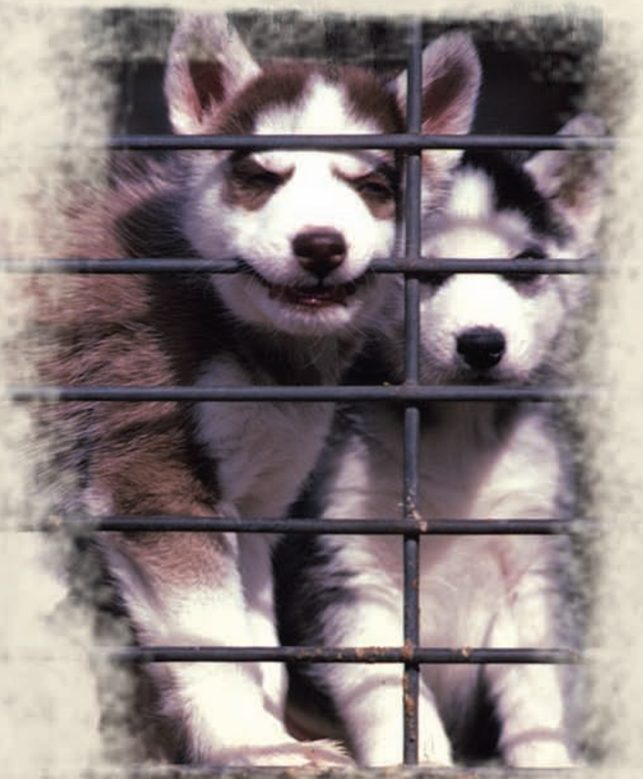

# Online Shopping—the New Face of a Terrible Business

You think you've found the perfect breeders, with a website filled with pictures of cute puppies, claims of how much they adore their "furry babies," and warnings that they only sell to "qualified homes." Everything feels right about this place, but beware—such websites are one of the newest scams puppy mills are running, and there's no way to know by looking at a website or talking to someone over the phone whether you're dealing with a puppy mill.

Websites allow puppy millers to "cut out the middleman" by selling directly to consumers. Not only is this more profitable, but in most states it allows the puppy mill to avoid being inspected by government agencies.

Websites loaded with pictures and promises of a loving home may seem like an ideal spot to find the right puppy for you, but remember that puppy mills house dogs in deplorable conditions, and they churn out puppies for quick sale and shipment. Your purchase could be supporting cruelty.

## Heart Set on a Purebred Dog?

Shelters and breed rescue groups have purebred dogs available for adoption every day. Contact The Humane Society of the United States Companion Animals staff at 202-452-1100 or 2100 L Street, NW, Washington, DC 20037, or visit **humanesociety.org/puppy** for help finding a purebred rescue group or shelter in your area.

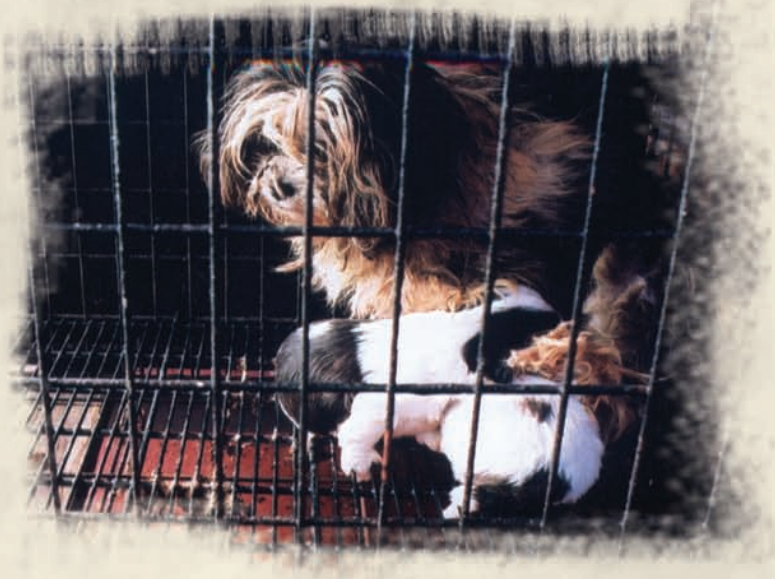

**For more on puppy mills, and for help finding a breeder who isn't running a puppy mill, visit humanesociety.org/puppy.**

Celebrating Animals | Confronting Cruelty

THE HUMANE SOCIETY OF THE UNITED STATES

2100 L Street, NW Washington, DC 20037
humanesociety.org

©2008 The HSUS. All rights reserved. Printed on 100% post-consumer recycled paper, processed chlorine free, with soy-based ink.

## 2.2 Qualtrics Survey

# Leafletting Follow up Survey

Start of Block: Instruction

Note
**Compensation**: When you complete this survey, you will be entered in to a drawing for a **$50 Amazon.com Gift Card**. There are 11 gift cards for [redacted] students. If you are selected, the gift card will be emailed to you.

Note: Please be as truthful as possible and answer to the best of your knowledge. Your responses will be used solely for research purposes. As such, we would like your honest answers. All responses will be kept strictly confidential.

End of Block: Instruction

Start of Block: Survey Questions

Q1 Which of these describes your current diet? Pick all that apply

- ▢ Ketogenic diet (high fat, low-carb diet) (1)
- ▢ Atkins Diet (eat low carbohydrate, high protein foods) (2)
- ▢ Paleolithic Diet (consists of fish, meats, eggs, vegetables, fruit, fungi, roots, and nuts) (3)
- ▢ Mediterranean Diet (A diet mimicking the traditional dietary patterns of southern Italy) (4)
- ▢ Pescatarian Diet (eat fish, egg, and milk products, but no other meat (including chicken)) (5)
- ▢ Vegetarian Diet (eat egg and milk products, but no meat (including fish or chicken)) (6)
- ▢ Vegan Diet (eat no meat (including fish or chicken), milk products, egg, or other animal products) (7)
- ▢ Meat Reduction Diet (A diet reducing meat consumption, for example Meatless Mondays) (8)
- ▢ No specific diet (A diet with no specific preferences or exclusions) (9)
- ▢ Other (10) ________________________________________________

---

Q2 Has your diet changed over the last month?

- ○ Yes (1)
- ○ No (2)
- ○ Other (3) ________________________________________________

---

*Display This Question:*

*If Has your diet changed over the last month? = Yes*

Q3 If your diet changed over the last month, which of the following are reasons you think contributed to the change? (check all that apply)

- ☐ Health reasons (my idea) (1)
- ☐ Health reasons (doctor's suggestion) (2)
- ☐ Allergies (3)
- ☐ Environmental reasons (4)
- ☐ Animal cruelty (5)
- ☐ Social justice (6)
- ☐ Religious reasons (7)
- ☐ Ethical reasons (8)
- ☐ Like the taste better (9)
- ☐ Cost (10)
- ☐ Convenience (11)
- ☐ Changed where I get food or who prepares food for me because of other life changes (12)
- ☐ Seasonal variation (13)
- ☐ Other (14) ________________________________________________
- ☐ Don't know (15)
- ☐ Not applicable: I did not change my diet (16)

Page Break

Q4 Did a leafleter give you any of these leaflets in the past month (this semester)? Check all that apply.

- ☐ A (1)
- ☐ B (2)
- ☐ C (3)
- ☐ D (4)
- ☐ E (5)
- ☐ None (6)
- ☐ Not sure (7)

---

*Display This Question:*

*If Did a leafleter give you any of these leaflets in the past month (this semester)? Check all that... = A*

*Or Did a leafleter give you any of these leaflets in the past month (this semester)? Check all that... = B*

*Or Did a leafleter give you any of these leaflets in the past month (this semester)? Check all that... = C*

*Or Did a leafleter give you any of these leaflets in the past month (this semester)? Check all that... = D*

*Or Did a leafleter give you any of these leaflets in the past month (this semester)? Check all that... = E*

Q5 If so, did you read it?

- ◯ Yes, all of it (1)
- ◯ Yes, some of it (2)
- ◯ I glanced at it (3)
- ◯ No, I did not (4)

*Display This Question:*

*If Did a leafleter give you any of these leaflets in the past month (this semester)? Check all that... = A*

*Or Did a leafleter give you any of these leaflets in the past month (this semester)? Check all that... = B*

*Or Did a leafleter give you any of these leaflets in the past month (this semester)? Check all that... = C*

*Or Did a leafleter give you any of these leaflets in the past month (this semester)? Check all that... = D*

*Or Did a leafleter give you any of these leaflets in the past month (this semester)? Check all that... = E*

Q6 If a leafleter gave you leaflet(s), do you think the leaflet(s) affected you?

- ◯ Yes, I am eating differently. (1)
- ◯ Yes, I am eating the same but am thinking differently about farming practices. (2)
- ◯ Yes, I am thinking differently about pet cats and dogs. (3)
- ◯ Yes, in a different way (please elaborate) (4) ____________________
- ◯ Maybe a little. (5)
- ◯ No. (6)

*Display This Question:*

*If If so, did you read it? = Yes, all of it*

*Or If so, did you read it? = Yes, some of it*

*Or If so, did you read it? = I glanced at it*

Q7 Reading the leaflet(s) taught me about (choose all the reasons that apply)

- ☐ The treatment of animals in farms. (1)
- ☐ The climate-change impact of my diet (3)
- ☐ How animals enjoy music. (2)
- ☐ Pet adoption and puppy mills. (4)
- ☐ I knew most of the information in the leaflet already. (6)

---

*Display This Question:*

*If If so, did you read it? = Yes, all of it*

*Or If so, did you read it? = Yes, some of it*

*Or If so, did you read it? = I glanced at it*

Q8 After reading the leaflet I thought more about (choose all the reasons that apply)

- ☐ The treatment of animals in farms. (2)
- ☐ The climate-change impact of my diet (4)
- ☐ How animals enjoy music. (5)
- ☐ Pet adoption and puppy mills. (3)
- ☐ I did not think more about any of the above issues. (6)

---

Q9 Did you read any of the other leaflets displayed? Perhaps a friend had one and showed you, or you found one and read it.

- ☐ A (1)
- ☐ B (2)
- ☐ C (3)
- ☐ D (4)
- ☐ E (5)
- ☐ None (6)
- ☐ Not sure (7)

---

Page Break

Q10 In your view how important is the issue of mistreatment of farm animals?

- Not at all important (1)
- Moderately important (2)
- Extremely important (3)
- I don't know (4)

---

Q11 In your view how impactful is personal choice of food items on how farm animals are treated?

- Not very impactful (1)
- Moderately impactful (2)
- Extremely impactful (3)
- I don’t know (4)

---

Q12 In your view how accessible are animal product replacements (alternative plant-based products)?

- Not accessible (1)
- Moderately accessible (2)
- Easily accessible (3)
- I don’t know (4)

---

Q13 How willing are you to make lifestyle changes to help reduce mistreatment of farm animals?

- ○ Not willing to make **any** lifestyle changes (1)
- ○ Willing to make **small** lifestyle changes (2)
- ○ Willing to make **moderate** lifestyle changes (3)
- ○ Willing to make **big** lifestyle changes (4)

**End of Block: Survey Questions**

---

**Start of Block: Demographic Information**

Q14 Are you of Hispanic, Latino, or Spanish origin?

- ○ Yes (1)
- ○ No (2)

---

Q15 How would you identify yourself?

- ▢ American Indian or Alaska Native (1)
- ▢ Asian (2)
- ▢ Black or African American (3)
- ▢ Native Hawaiian or Other Pacific Islander (4)
- ▢ White (5)
- ▢ Other (if so, please write in) (6) ________________________________________________

---

Q16 What is your gender?

- ○ Male (1)
- ○ Female (2)
- ○ Other/Non-binary/Decline to state (3)

---

Q17 Are you first-generation in your family to attend college?

- ○ Yes (1)
- ○ No (2)

End of Block: Demographic Information

## 3 Pre-Analysis Plan (PAP)

# Effectiveness of informational pamphlets

# Outline of Analysis

1. **Structure of data**
    a. **Food data**
        i. Observation is a purchase at the dining hall.
        ii. Date and Timestamp
        iii. Itemized food items
        iv. Meat or not (beef, poultry, veg, salad and fish)
        v. Purchases on Fridays, mornings and weekends are omitted (food items are not clearly identifiable for these purchases).
    b. **Survey data**
        i. Gender
        ii. Race
        iii. Self-identified diet
        iv. Memory recall about the intervention tools
        v. Persuasivity of intervention
        vi. Informativeness of intervention
        vii. View towards treatment of farm animals
        viii. View towards impact of personal choice
        ix. Self-reported attempted diet change
        x. Reasons for changing/not changing
2. **Variables**
    a. **Outcomes**
        i. Main Outcome: Purchase was meat or not
        ii. Survey Outcomes
            1. Diet changed
            2. Reason for diet change
            3. Remembered leaflet
            4. Read the leaflet
            5. Flyer affected you?
            6. Importance of animal, personal choice, accessibility of replacements, willing to make changes
    b. **Regressors**
        i. Treatment
        ii. Leafletting variables: day, leafletter, hour
        iii. Survey variables:
            1. Current diet.
            2. Has diet changed?
            3. Reasons for diet change
            4. Leaflet: Remembered the leaflet, read the leaflet, leaflet affected you?, taught me, persuaded me, increased my motivation

5. Importance of animal, personal choice, accessibility of replacements, willing to make changes. Index of these variables.
6. Race/Ethnicity, Gender, First Generation

iv. FE: Individual, meal

**3. Statistical Model**

**a. Descriptive summary statistics**

**b. Main Regression**

$Y_{im} = \theta_i + \lambda_m + \beta T_{im} + u_{im}$

Where: i represents the individual.
m is the meal (e.g. lunch on Oct 3).
$\theta$ & $\lambda$ represent individual and meal-specific FE respectively.
T is the treatment and u is the error term.

- We estimate $\beta$ using logit regression with individual FE using the whole year worth of purchase data.

**c. Survey regression**

$SY^j_i = \theta_i + \lambda_m + \beta_1 T_i + \beta_2 LV_i + \beta_3 SV_i + u_i$

Where: $SY^j$ represents survey outcomes;
$SY^1$ - Diet changed
$SY^2$ - Reason for diet change
$SY^3$ - Remembered leaflet
$SY^4$ - Read the leaflet
$SY^5$ - Flyer affected you?
$SY^6$ - Importance of animal, personal choice, accessibility of replacements, willing to make changes

$LV_i$ represent the leaflet variables;
$day_i$ -Day
$leaf_i$ - Leafletter
$hr_i$ - Hour

$SV_i$ represents survey variables;
$cdiet_i$ - Current diet.
$hasdc_i$ - Has diet changed?
$reasons_i$ - Reasons for diet change
$Leaflet_i$ - Leaflet: Remembered the leaflet, read the leaflet, leaflet affected you? taught me, persuaded me, increased my motivation.
$index_i$ - Importance of animal, personal choice, accessibility of replacements, willing to make changes. Index of these variables.
$race_i$ - Race/Ethnicity
$gender_i$ – Gender
$firstgen_i$ - First Generation

- We estimate β's using logit and multinomial logit regression depending on the type of the survey outcome variable.

**d. Heterogeneous treatment effects**

$Y_{im} = \theta_i + \lambda_m + \beta_1 T_{im} + \beta_2 T_{im} X \quad + \beta_3 T_{im} X\ race_i + \ldots + u_{im}$

- Estimate β's using logit regression

**e. Mechanisms**

$Y_{im} = \theta_i + \lambda_m + \beta_1 T_{im} + \beta_2 T_{im} X\ Leaflet_i + \ldots + u_{im}$

$Y_{im} = \theta_i + \lambda_m + \beta_1 T_{im} + \beta_2 T_{im} X\ remember_i + \beta_3 T_{im} X\ read_i + \beta_4 T_{im} X\ affectedme_i + \beta_5 T_{im} X\ taughtme_i + \beta_6 T_{im} X\ persuadedme_i + \beta_7 T_{im} X\ motivatedme_i + \ldots + u_{im}$

- Estimate β's using logit regression
